# Genome-Wide Identification and Expression Analysis of the Thioredoxin (*Trx*) Gene Family Reveals Its Role in Leaf Rust Resistance in Wheat (*Triticum aestivum* L.)

**DOI:** 10.3389/fgene.2022.836030

**Published:** 2022-03-25

**Authors:** Ramesh Bhurta, Deepak T. Hurali, Sandhya Tyagi, Lekshmy Sathee, Sandeep Adavi B, Dalveer Singh, Niharika Mallick, Viswanathan Chinnusamy, Vinod, Shailendra K. Jha

**Affiliations:** ^1^ Division of Genetics, ICAR-IARI, New Delhi, India; ^2^ Division of Plant Physiology, ICAR-IARI, New Delhi, India

**Keywords:** wheat, leaf rust, thioredoxin, reactive oxygen species (ROS), genome-wide analysis

## Abstract

Bread wheat (*Triticum aestivum* L.; *Ta*) is the staple cereal crop for the majority of the world’s population. Leaf rust disease caused by the obligate fungal pathogen, *Puccinia triticina* L., is a biotrophic pathogen causing significant economic yield damage. The alteration in the redox homeostasis of the cell caused by various kinds of reactive oxygen species (ROS) and reactive nitrogen species (RNS) in response to pathogenic infections is controlled by redox regulators. Thioredoxin (Trx) is one of the redox regulators with low molecular weight and is thermostable. Through a genome-wide approach, forty-two (42) wheat *Trx* genes (*TaTrx*) were identified across the wheat chromosome groups A, B, and D genomes containing 12, 16, and 14 *Trx* genes, respectively. Based on *in silico* expression analysis, 15 *TaTrx* genes were selected and utilized for further experimentation. These 15 genes were clustered into six groups by phylogenetic analysis. MicroRNA (miRNA) target analysis revealed eight different miRNA-targeted *TaTrx* genes. Protein–protein interaction (PPI) analysis showed *TaTrx* proteins interact with thioredoxin reductase, peroxiredoxin, and uncharacterized proteins. Expression profiles resulting from quantitative real-time PCR (qRT-PCR) revealed four *TaTrx* genes (*TaTrx11-5A*, *TaTrx13-5B*, *TaTrx14-5D*, and *TaTrx15-3B*) were significantly induced in response to leaf rust infection. Localization of ROS and its content estimation and an assay of antioxidant enzymes and expression analysis suggested that *Trx* have been involved in ROS homeostasis at span 24HAI-72HAI during the leaf rust resistance.

## Introduction

Wheat (*Triticum aestivum* L.) leaf rust disease caused by *Puccinia triticina* L. is the most prevalent disease among the three rust-related diseases. About 50% yield reduction has been reported when conditions are favorable for leaf rust infection and depending on the plant’s developmental stage at disease (Huerta-Espino et al., 2011; Dakouri et al., 2013). Though the damage caused by rust can be minimized through chemicals, host resistance is the most economical and sustainable way to cope with this disease. So far, eighty (80) leaf rust resistance genes have been identified and designated, and different *Lr* genes (APR and SR) are being used in different wheat breeding programs to develop rust-resistant varieties (Tomar et al., 2014; Kumar et al., 2021).

A strategy for manipulating resistance can be developed by understanding the pathways involved in defense signaling. Plants develop and execute various multifaceted sensory mechanisms to recognize a particular pathogen attack and work against it to maximize plant survival (Lamers et al., 2020). These molecular mechanisms are well elucidated and explained to a great depth (Wang et al., 2019). The impact of thioredoxin (Trx) and reactive oxygen species (ROS) as a critical factor in the plant system against biotic stress responses is one area to understand and exploit its role in resistance. Regulations of cysteine modification depend on Trx activity. Thiol-based redox modification of Trxs alters the activity of target proteins involved in the interaction network (Rouhier et al., 2015). Reactive oxygen species and oxidative stress produced by metabolism, respiration, and defense responses are sensed by thioredoxins, and the downstream target signaling proteins are modulated to regain redox balance (He et al., 2017).

Thioredoxins (Trxs) are heat-stable multifunctional ubiquitous proteins that act as oxidoreductase and are involved in various metabolic processes such as photosynthesis and response to abiotic and biotic stresses, depending on their inherent biochemical properties (Holmgren, 1968; Wong, 2002). Thioredoxins are also confirmed to be involved in the orchestration of Systemic Acquired Resistance (SAR) in plants by its redox activity that converts the oligomeric form of the nonexpressor of pathogenesis-related gene1 (NPR1), a master regulator of SAR, into a monomeric form in *Arabidopsis* (Fu et al., 2012; Moreau et al., 2012). Trxs possess a characteristic conserved active site consisting of WCGPC residues that catalyze thiodisulfide bridges (Collet et al., 2003). Trx proteins exhibit numerous variants, and in plants, they are classified as Trxf, Trxm, Trxh, Trxo, Trxx, and Trxy isoforms (Gelhaye et al., 2004). Apart from their prominent role in the redox-based modification of proteins that are involved in resistance response, thioredoxins are also associated with various biochemical processes, viz., enzyme regulation (Holmgren, 1989), modulation of transcription factor (Schenk et al., 1994), as the donor of hydrogen (Holmgren, 1989), protection against oxidative stress (Chae et al., 1994), and in-plant resistance (Mata-Pérez and Spoel, 2019).

The role of ROS/RNS is well known as an essential mediator of redox signaling in defense pathways (Apel and Hirt, 2004; Baxter et al., 2014; Mittler, 2002; Noctor et al., 2018; Mittler et al., 2004; Torres et al., 2006). The activation of the plant resistance responses against a pathogen with the rapid production of an enormous amount of ROS and reactive nitrogen species (RNS) that results in alteration of redox homeostasis of the cell (Mata-Pérez and Spoel, 2019). Plants induce a defense response against pathogen attack via inducing pathogenesis-related (PR) genes and localized cell death at the infection site, collectively known as the hypersensitive response (HR). Further ROS is generated after pathogen recognition in various subcellular compartments responsible for the orchestration of HR (Zubriggen et al., 2010). Wang et al. (2020) demonstrated that ROS (superoxide radicals and H_2_O_2_) is generated during the interaction of leaf rust, *Puccinia triticina* (Pt) and wheat. When a pathogen attacks the plant, the plant–microbe interaction is sensed by membrane receptors, and an oxidative (ROS) burst occurs in the apoplast by activating NADPH oxidases (NOX/RHOH) (Rahikainen et al., 2016). Furthermore, ROS signaling is also required to induce immunity-related genes (de Torres Zabala et al., 2015).

Cellular ROS homeostasis involves several enzymatic and non-enzymatic antioxidant systems in plants (Lu and Holmgren, 2014; Considine and Foyer, 2020). Among these antioxidant systems, Trx modulates ROS scavenging and actively regulates cellular redox homeostasis. It was reported that functional loss of Trx leads to altered levels of ROS (Vieira Dos Santos and Rey, 2006; Schmidt et al., 2016). For instance, thioredoxin genes *Trxh8* and *Trxh5* are highly induced in response to abiotic and biotic stresses in rice (Tsukamoto et al., 2005). A novel cis-element regulates three antioxidant genes *Trxh*, *Grx*, and *SOD* in rice (Tsukamoto et al., 2005). These studies advocate the coordinated participation of thioredoxin and other antioxidants in cellular defense against high levels of ROS. In the recent past, significant advancements have been made in understanding the pathway of defense responses against rust pathogen, and new dimensions are expected to be added in the future. However, to our best knowledge, change in the expression profile of *Trx* genes against leaf rust resistance in wheat and its relation with *ROS* have not been studied yet. Therefore, based on the importance of Trx proteins in biotic stress response and ROS balance during plant–pathogen interaction, we conducted 1) genome-wide identification and *in silico* analysis of *Trx* family genes in wheat and 2) expression profiling of selected *TaTrx* family genes in response to leaf rust infection in wheat.

## Materials and Methods

### *In Silico* Chromosome Localization of Wheat *Trx* Genes

The amino acid sequences of Trx proteins from *Arabidopsis (AtTrxh3; At5G42980)*) and rice (*OsTrxh5*; *Os07T0190800)* were extracted from TAIR (https://www.arabidopsis.org/index.jsp), and The Rice Annotation Project database rap-db (https://rapdb.dna.affrc.go.jp). The hidden Markov model (HMM) profile of the Trx domain (PF00085.22) and (PF00085.22) was used as a query to identify the wheat Trx domain in the wheat genome database using the software HMMER (http://pfam.sanger.ac.uk. A BLAST algorithm was also used to identify the putative *Trx* genes present across the wheat genome. The amino acid sequences of *Trx* candidate genes from *Arabidopsis* and rice were used *OsTrxh5*, *Os07T0190800* as a query against the fully annotated genome sequence of wheat accessible at Ensembl Plants release 47 (https://plants.ensembl.org/index.html). All non-redundant sequences with E-values < 1.0^E-05^ were identified and selected. Protein, CDS, cDNA, and genomic sequences of all the chosen IDs were downloaded for further analysis. All the identified sequences were further analyzed to search conserved domains using secondary databases, including InterPro (https://www.ebi.ac.uk/interpro) and PROSITE (https://prosite.expasy.org). To determine the conserved or diverse *Trx* genes among the different wheat species, a BlastP search was also conducted using the genome databases. This analysis used the sequence release 52 of three wheat species *T. Urartu* (A genome), *T. turgidum* (AB genome), and *A. tauschii* (D Genome) available on Ensembl Plants. All the identified wheat *Trx* genes (*TaTrx*) IWGSC IDs screened against information available in public repositories, including IWGSC-URGI (https://wheat-urgi.versailles.inra.fr/) and EnsemblPlants (Bolser et al., 2016) and were *in silico* mapped on seven homeologous chromosome groups accordingly.

### Physiochemical Properties and Subcellular Localization of Trx Protein

The amino acid sequences of all the identified Trx proteins retrieved from Ensembl Plants were analyzed using the ProtParam tool (Expasy website: https://web.expasy.org/protparam/) (Gasteiger et al., 2005) to obtain different physiochemical properties like the grand average of hydropathicity (GRAVY), isoelectric point (IP), aliphatic index (Ai), molecular weight (Mw), and instability index (Ii). Further, BUSCA (http://busca.biocomp.unibo.it/) (Savojardo et al., 2018), a web server, was used to predict the subcellular location of candidate Trx proteins.

### Gene Structure, Conserved Motif Identification, and Evolutionary Analysis

Both cDNA and genomic sequences were analyzed in GSDS v2.0 (Gene Structure Display Server) (Hu et al., 2015). The regulatory motif variation in identified *TaTrx* genes was analyzed using the amino acid sequence of respective *TaTrx* genes in MEME 5.0.5 (Bailey et al., 2009) with selection criteria set at 15 AA, minimum length of 6, and a maximum length of 50 amino acids (Bailey et al., 1994). Phylogenetic analysis was performed using the identified amino acid sequences of *TaTrxs* and the *Trxs* earlier reported in *Arabidopsis* and rice. For this purpose, protein sequences of *Arabidopsis* (22 Trxs) and rice (28 Trxs) were downloaded from TAIR (https://www.arabidopsis.org/) and rap-db (https://rapdb.dna.affrc.go.jp/), respectively. All the collected amino acid sequences of identified *Trx* genes were aligned with ClustalW (http://ebi.ac.uk/Tools/msa/clustalW2). The aligned output file was used by the software MEGA7.0 (Kumar et al., 2016) to establish evolutionary relationship among extracted all *Trx* proteins. MEGA 7.0 constructed a phylogenetic tree using the maximum likelihood method (Kumar et al., 2016) with the substitution model, uniform rates, and pair-wise deletion with bootstrap values for 1,000 iterations calculated and expressed as percentages (Felsenstein, 1985).

### Putative Cis-Regulatory Element Identification in the Promoter Region of the *TaTrx* Genes

The 1,000 bp *TaTrx* gene sequences upstream to the start codon were retrieved from Ensembl Plants. *TaTrx* sequences were analyzed in the PlantCARE database (http://bioinformatics.psb.ugent.be/webtools/plantcare/html/) to scan cis-regulatory elements (Lescot et al., 2002). These 1,000 bp upstream sequences were investigated to identify the cis-regulatory elements to hormone responsiveness (involved in stress) and defense and stress responsiveness.

### Potential miRNA Target for *TaTrx* Genes

Prediction of potential miRNA target(s) for all the identified *TaTrx* genes were made by aligning the mRNA sequence against the miRNAs in wheat with perfect or near-perfect complementarity to mRNA using the psRNATarget server with default parameters (Dai X. et al., 2018). The interaction network of potential miRNA and *Trx* genes in wheat was predicted using Cytoscape (Dai L. et al., 2018).

### Homology Modeling and 3D Structure Analysis of TaTrx Proteins

To compare the three-dimensional (3D) structure of TaTrx proteins, Homology modeling (comparative modeling) was done using the SWISS server (https://swissmodel.expasy.org/) to compare the three-dimensional (3D) structure of TaTrx proteins. Position-specific iterated BLAST (PSI-BLAST) was done to align template and target proteins; homolog template was selected based on similarity from protein database (PDB). The 3D structure of TaTrx proteins was predicted by an automated SWISS-MODEL server (https://swissmodel.expasy.org) (Waterhouse et al., 2018) and was further visualized in UCSF CHIMERA (Pettersen et al., 2004). TaTrx proteins were compared against templates 3d22.1.A, 2iwt.1.A, 2vlt.1.A, 2vm1.3.A, 1fb0.1.A, and 1faa.1.A from PDB. Ramachandran plot, per cent sequence identity of the target TaTrx proteins with template protein, template protein ID, QMEAN, template description, oligo state, and Ramachandran favored per cent were analyzed using the SWISS server.

### Analysis of Protein–Protein Interaction Network

Interaction of TaTrx proteins with other proteins was analyzed using amino acid sequences of respective TaTrx proteins in STRING v11.0 (https://string-db.org) (Szklarczyk et al., 2019), the protein with the highest bit score was considered for analysis. GeneMANIA server (Warde-Farley et al., 2010) was also used for protein–protein interaction analysis by utilizing *Arabidopsis* interactome data.

### *In Silico* Expression Analysis

Wheat RNA transcriptome data [WheatEXP (http://www.wheat-expression.com/)] available under different biotic stress (Powdery mildew pathogen E09 and stripe rust pathogen CYR31) treatments were used to compare the relative expression of all the identified *TaTrx* genes. Extracted transcript IDs were used for generating a heat map using the Wheat Expression browser expVIP (http://www.wheat-expression.com).

### Plant Materials and Pathogen

The seeds of two contrasting wheat genotypes, Chinese spring (CS; susceptible genotype) and Transfer (TR; resistant genotype), an Introgression line of leaf rust resistance gene (*Lr9*) in CS background available at the Division of Genetics, IARI New Delhi, were used for the current experiment. The uredospores of leaf rust pathogen *Puccinia triticina* Eriks Pathotype 77-5 (121-R-63) were used to inoculate two wheat genotypes.

### Inoculation and Sampling

Seedlings of CS and TR were grown in the National Phytotron Facility at ICAR-Indian Agricultural Research Institute, New Delhi, following the protocol by Prabhu et al. (2012). Ten to fifteen seeds of each genotype were sown, raised, and seedlings at the two-leaf stage were inoculated with the pathotype 77-5 using the protocol by Dhariwal et al. (2011). After inoculation, seedlings were kept in a humidity chamber for 24 h. Sampling was done by collecting the leaves at 0 HAI (Hours After Inoculation, uninoculated), 24 HAI, 72 HAI, and 144 HAI for analysis.

### Isolation of Total RNA and cDNA Preparation

Total RNA was extracted from sampled leaf tissues (3 biological and 3 technical replicates) of control and treated plants at different intervals after leaf rust infection using RNAeasy kit (Qiagen Inc., Chatsworth CA 91311, United States, Cat No: 749040) as per the protocol and instruction described in the kit. RNA was quantified using a Thermo nanodrop spectrophotometer, and the purity was analyzed by checking the values of the ratio of A_260_/A_280_ and using gel electrophoresis. The cDNA was prepared using Invitrogen, Life Technologies, United States, reverse transcriptase kit.

### Primer Designing and qRT-PCR Analysis

cDNA sequences of all the identified *TaTrx* genes were used to design primers (Supplementary Table S1). The qRT-PCR assay was performed to determine the expression level of *Trx* genes in wheat. The qRT-PCR assay was done using a mixture of cDNA, forward primer, reverse primer, and SYBR^∗^ Green Master Mix (Applied Biosystems, United States) on a real-time detection system (CFX96 Touch real-time PCR detection, BIO-RAD life sciences). To set up qRT-PCR, all components of cDNA, forward and reverse primer, and SYBR^∗^ Green were carefully added in PCR plates and centrifuged for 1–2 min. All components were thawed properly to avoid differences in concentration. All the above steps were performed on ice. Care was taken to prevent the exposure of SYBR^∗^ Green to light and high temperatures. A master mix includes 4.5 µl RNase-free water, 0.75 µl forward primer, 0.75 µl reverse primer, and 7.5 µl Power SYBR Green Master Mix. 1.5 µl cDNA template was pipetted out into the PCR plate followed by the master mix. The master mix was kept in the dark to avoid light exposure. Wheat gene *TaActin* (housekeeping gene) was used as an internal control for normalization of the data for each transcript (Sathee et al., 2018) and level of expression or fold change in expression of *TaTrx* genes were analyzed using the 2^−ddCt^ method (Livak and Schmittgen., 2001).

### Estimation of Reactive Oxygen Species

Based on previous reports on ROS and RNS abundance (Qiao et al., 2015), 72 HAI samples were selected to study ROS localization and the activity of antioxidant enzymes. After inoculation with pathotype 77-5, the leaves of the two genotypes were sampled 72 h after inoculation for ROS staining, ROS determination, and for an assay of antioxidant enzymes. The second leaves of inoculated and uninoculated wheat seedlings were used to analyze tissue localization of superoxide ions (O^2−^) anions and H_2_O_2_. For determining O^2−^ radicals, 1 cm long leaf cuttings were incubated in a 6 mM NBT solution prepared in sodium citrate buffer (pH 7.5). The samples were vacuum infiltrated at 60 KPa for 10 min. After 10 min at room temperature, the samples were boiled in 80% ethanol in boiling water until the tissue turned translucent. Samples were mounted on glass slides after dipping in 20% glycerol. The formation of the dark blue color specified the localization of O^2−^ radicals. For detecting the formation of H_2_O_2_, the leaf cuttings were incubated in 1 mg/ml (p^H^ 3.8) DAB (3,3′-Diaminobenzidine) solution. Vacuum infiltration and chlorophyll removal steps were followed as described previously. The formation of the brown color specified the localization of H_2_O_2_. The slides were observed under stereomicroscope (EVOS XL Core), and the images were captured following Kumar et al. (2014).

### Assay of Oxidative Stress

Spectrophotometric assay quantitatively estimated superoxide radicals at 72HAI in fresh leaf tissue (Chaitanya and Naithani, 1994). The amount of NBT reduced by O^2−^ radicals was assayed. Phosphate buffer of 0.2 M (pre-cooled, *p*H 7.2) was used to homogenize the leaf sample (1 g). The homogenized sample was centrifuged at 10000 g for 30 min at 4°C. The supernatant was collected and stored in a −20°C freezer until further analysis. The assay mixture comprised 0.1 mM EDTA, 0.075 mM NBT, 13.33 mM l-methionine, 25 mM Na_2_CO_3,_ 250 µl of supernatant in a final volume of 3 ml, and the absorbance was measured at 540 nm.

For estimation of H_2_O_2_, 1 g of leaf sample was crushed in liquid nitrogen, homogenized in 10 ml cooled acetone, and filtered (Whatman no. 1). The filtrate was mixed with 5 ml ammonium solution and 4 ml titanium reagent for precipitation of titanium-hydrogen peroxide complex. The precipitate obtained after centrifugation at 10000 g for 10 min was dissolved in 10 ml of 2M H_2_SO_4_. This dissolved precipitate was re-centrifuged, and the supernatant was used for spectroscopic absorbance measurement at 415 nm (Rao et al., 1997). The membrane injury was estimated by Evans blue staining followed by spectrophotometric estimation of tissue-bound dye (Kato et al., 2007).

### Estimation of Antioxidant Enzymes

One gram of fresh leaf sample was sampled for preparation of extract to measure the activity of ascorbate peroxidase (APX), superoxide dismutase (SOD), peroxidase (POX) and catalase (CAT) enzymes. The sample was homogenized in 10 ml of 0.1 M phosphate extraction buffer (pH 7.5, comprising 1 mM ascorbic acid and 0.5 mM EDTA). The extract was filtered using a four-layered cheesecloth. The filtrate obtained was centrifuged at 15,000 g for 20 min. The activity of enzymes was estimated using the supernatant (Dhindsa et al., 1981).

Inhibition of the photochemical reduction of NBT was the basis for SOD activity estimation (Dhindsa et al., 1981). From 2 mM riboflavin stock, 0.1 ml was mixed into the reaction mixture containing 0.1 ml enzyme extract and incubated for 15 min under 15W lamps. A set of reaction mixture kept in the dark (transparent) served as the blank. After 15 min of incubation, absorbance was recorded at 560 nm.

Ascorbic acid-induced reduction in absorbance (290 nm) was served as the basis for estimation of Ascorbate peroxidase activity (Nakano and Asada, 1981). The reaction mixture comprised of 1.5 ml of 100 mM potassium phosphate buffer, 0.5 ml 3.0 mM ascorbic acid, 0.1 ml of 3.0 mM of EDTA, 0.1 ml of 3.0 mM Hydrogen peroxide, 0.1 ml enzyme of enzyme extract, and final volume made to 3 ml using double distilled water. Before measuring the optical density (OD), 0.2 ml of hydrogen peroxide was added to the reaction mixture. The reduction in the OD was recorded for 1 min at 290 nm wavelength in a UV–visible spectrophotometer.

Reduction in absorbance (240 nm) due to hydrogen peroxide decomposition served as the basis for estimating catalase (CAT) activity. The reaction mixture (3 ml) includes 1.5 ml of 0.1 M phosphate buffer and 0.5 ml of 75 mM H_2_O_2_. To which diluted enzyme extract of 50 µl was added and UV–visible spectrophotometer was used to measure the decline in the absorbance for 1 min at 240 nm.

The rise in absorbance (470 nm) due to the development of tetra-guaiacol served as the basis for estimating peroxidase enzyme activity. The reaction mixture includes enzyme extract (0.1 ml), guaiacol (16 mM), phosphate buffer (50 mM, pH 6.1), and 2 mM H_2_O_2_. The reaction started with the addition of guaiacol (16 mM). The rise in absorbance at 470 nm was recorded in a UV–visible spectrophotometer for 1 min after the addition of guaiacol (16 mM). Tetra-guaiacol extinction coefficient (ε = 26.6 mM^−1^ cm^−1^) was used to calculate the enzyme activity (Castillo et al., 1984).

### Statistical Analysis

GraphPad Prism version 8 (La Jolla, California, United States) was used to compute analysis of variance (ANOVA), correlation, and prepare graphs. A model showing thioredoxins (Trx) mediated regulation of incompatible interaction between wheat and leaf rust pathogen was created using **Inkscape** 0.92.3 (freely available at: https://inkscape.org/).

## Results

### Genome-Wide Identification of Trx Genes

Genome-wide analysis of orthologous *Trx* genes in wheat (*TaTrx*) lead to the identification of 42 *TaTrx* genes from IWGSC RefSeq assembly v1.0 (Supplementary Table S2). Out of 42 *TaTrx* genes, 10 *TaTrx* genes were mapped on genome A, 21 *TaTrx* genes were located on genome B, and 11 *TaTrx* genes were positioned on genome D. The identified *TaTrx* genes were named *TaTrx*-1 to *TaTrx*-42 based on their chromosomal positions. InterPro and PROSITE analysis resulted in a conserved Trx domain in all the identified TaTrx and Trx proteins sequences of Rice and Arabidopsis. Blast search against four different wheat species led to identifying a set of 10, 20, and 32, *Trx* genes in *A. tauschii (DD), T. urartu (AA)*, and *T. turgidum (AABB),* respectively. Ten *Trx* genes in *A. tauschii* are located on six different chromosomes (1D: 3, 2D:2, 3D:1, 4D: 1, 5D: 2, and 6D: 1). *T. turgidum* contains a set of 32 *Trx* genes over six different homeologous groups involving wheat genome A (1A:3, 2A:3, 3A:2, 4A:3, and 7A:1) and B (1B:6, 2B:3, 3B:4, and 5B:5) (Supplementary Table S3).

### *In Silico* Expression Analysis of *TaTrx* Genes

**FIGURE 1:**
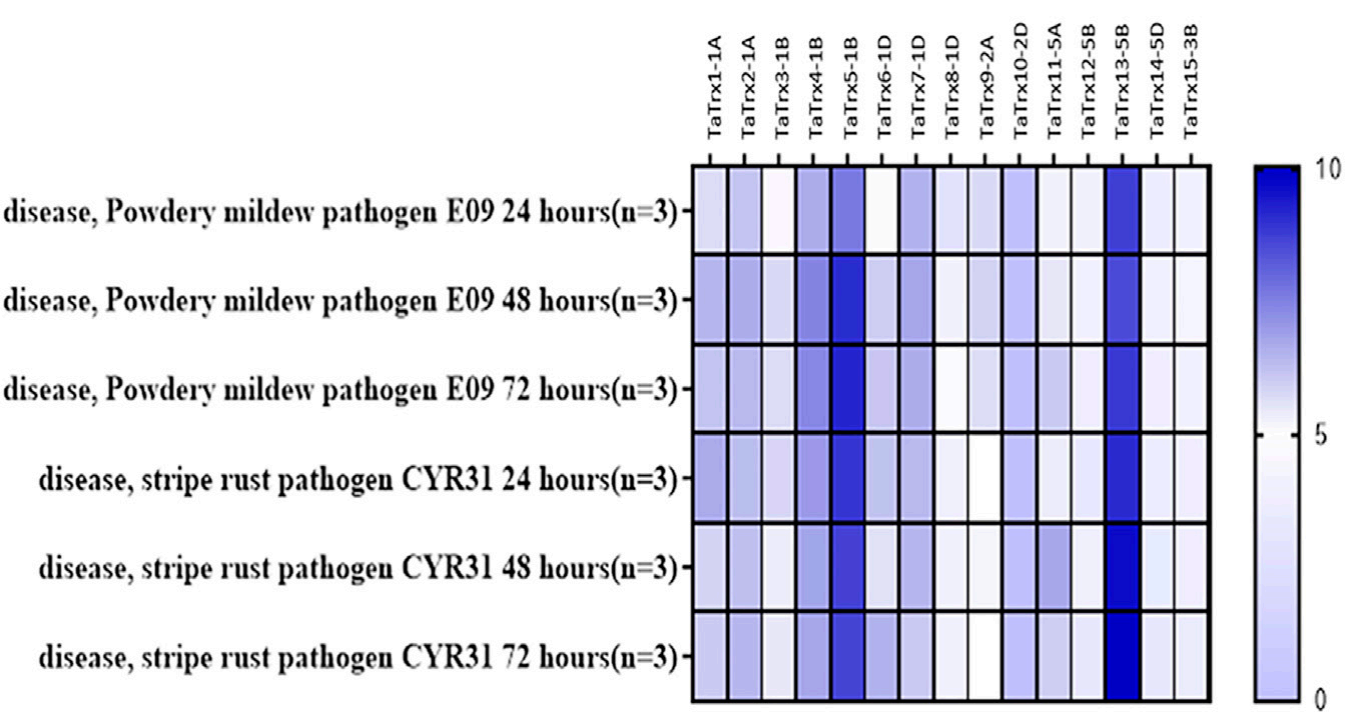
*In silico* expression analysis of TaTrx genes against biotic stresses using expVIP (http://wheat-expression.com/).

Expression of thioredoxins was retrieved from expVIP from experiments in wheat inoculated with stripe rust pathogen (Pathotype: CYR31) at the duration of 24 h, 48 h, and 72 h after inoculation (HAI) and powdery mildew pathogen (Pathotype: E09) at the span of 24 h, 48 h, and 72 h after inoculation. As leaf rust is also a biotrophic pathogen, we considered stripe rust’s *in silico* expression and powdery mildew (Figure 1). Based on the *in-silico* expression analysis in response to perturbance to the two biotrophic pathogens in the wheat expression browser, 15 selected *Trx* transcripts/genes showed a differential expression level to biotic perturbation. The Ensemble gene ID, gene length (bp), protein length (amino acid residues), number of splice variants and splice variant selected, coordinates, chromosomal location, and subcellular localization of *TaTrx* genes are listed in Table 1. Gene sequence investigation revealed that the length of coding sequence (CDS) of identified *TaTrx* genes range from 411 (*TaTrx10-2D*) to bp (*TaTrx13-5B*), and amino acids of corresponding protein range from 118 (*TaTrx9-2A*) to 189 (*TaTrx15-3B*).

**TABLE 1:** Details of selected TaTrx genes in wheat genome along with their subcellular locations.

S. no.	Gene	Ensemble ID	Splice variant	Coordinates	Length bp aa		Genome location	Subcellular location
1	*TaTrx1-1A*	TraesCS1A02G112400	1	114,060,494–114,063,211	858	131	1A: 114060494	Cytoplasm
2	*TaTrx2-1A*	TraesCS1A02G325600	1	516,430,393–516,432,298	845	130	1A: 516430393	Extracellular space
3	*TaTrx3-1B*	TraesCS1B02G132600	1	167,108,459–167,111,433	1,023	131	1B: 167108459	Cytoplasm
4	*TaTrx4-1B*	TraesCS1B02G338800	1	566,685,128–566,687,824	788	127	1B: 566685128	Chloroplast
5	*TaTrx5-1B*	TraesCS1B02G339000	1	566,823,400–566,831,663	742	188	1B: 566823400	Nucleus
6	*TaTrx6-1D*	TraesCS1D02G114000	1	110,052,054–110,054,117	664	131	1D: 110052054	Cytoplasm
7	*TaTrx7-1D*	TraesCS1D02G327200	1	419,471,916–419,473,737	757	126	1D: 419471916	Extracellular space
8	*TaTrx8-1D*	TraesCS1D02G327500	1	419,717,234–419,723,448	637	119	1D: 419717234	Nucleus
9	*TaTrx9-2A*	TraesCS2A02G243200	1	341,625,205–341,628,292	734	118	2A: 341625205	Cytoplasm
10	*TaTrx10-2D*	TraesCS2D02G244400	1	274,405,306–274,408,352	411	136	2D: 274405306	Cytoplasm
11	*TaTrx11-5A*	TraesCS5A02G448200	1	629,709,454–629,713,182	899	131	5A: 629709454	Nucleus
12	*TaTrx12-5B*	TraesCS5B02G111800	1	169,679,181–169,680,177	909	175	5B: 169679181	Mitochondrion
13	*TaTrx13-5B*	TraesCS5B02G452700	1	625,521,633–625,524,237	1,268	131	5B: 625521633	Nucleus
14	*TaTrx14-5D*	TraesCS5D02G454800	1	501,772,372–501,774,724	1,006	131	5D: 501772372	Nucleus
15	*TaTrx15-3B*	TraesCS3B02G453600	1	694,324,532–694,325,779	971	189	3B: 694324532	Chloroplast outer membrane

### Chromosomal Distribution

**FIGURE 2:**
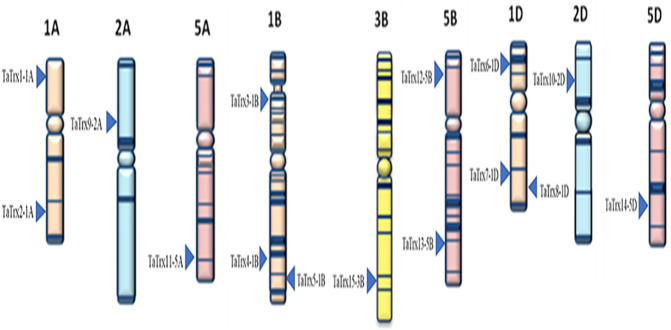
Distribution of the selected 15 TaTrx genes across the nine chromosomes of groups A, B, and D sub-genomes of wheat. Figure taken from the IWGSC website (https://www.wheatgenome.org/).

Based on the wheat genome database available at IWGSC-URGI and EnsemblPlants, positions of the selected *TaTrx* genes on corresponding chromosomes of A, B, and D genomes are shown in Figure 2. Selected 15 *TaTrx* genes were located on nine different wheat chromosomes (1A, 1B, 1D, 2A, 2D, 3B, 5A, 5B, and 5D). Out of 15 *TaTrx* genes, four genes were present on three chromosomes of the genome A (1A:2 genes, 2A:1gene and 5A:1gene), six *TaTrx* genes were located on three B genome chromosomes (1B:3 genes, 3B:1 gene, and 5B:2 genes), and five *TaTrx* genes were mapped on three D genome chromosomes (1D: 3 genes, 2D:1 gene, and 5D: 1 gene).

### Predicted Gene Structure, Phylogenetic Relationships, and Conserved Motif

**FIGURE 3:**
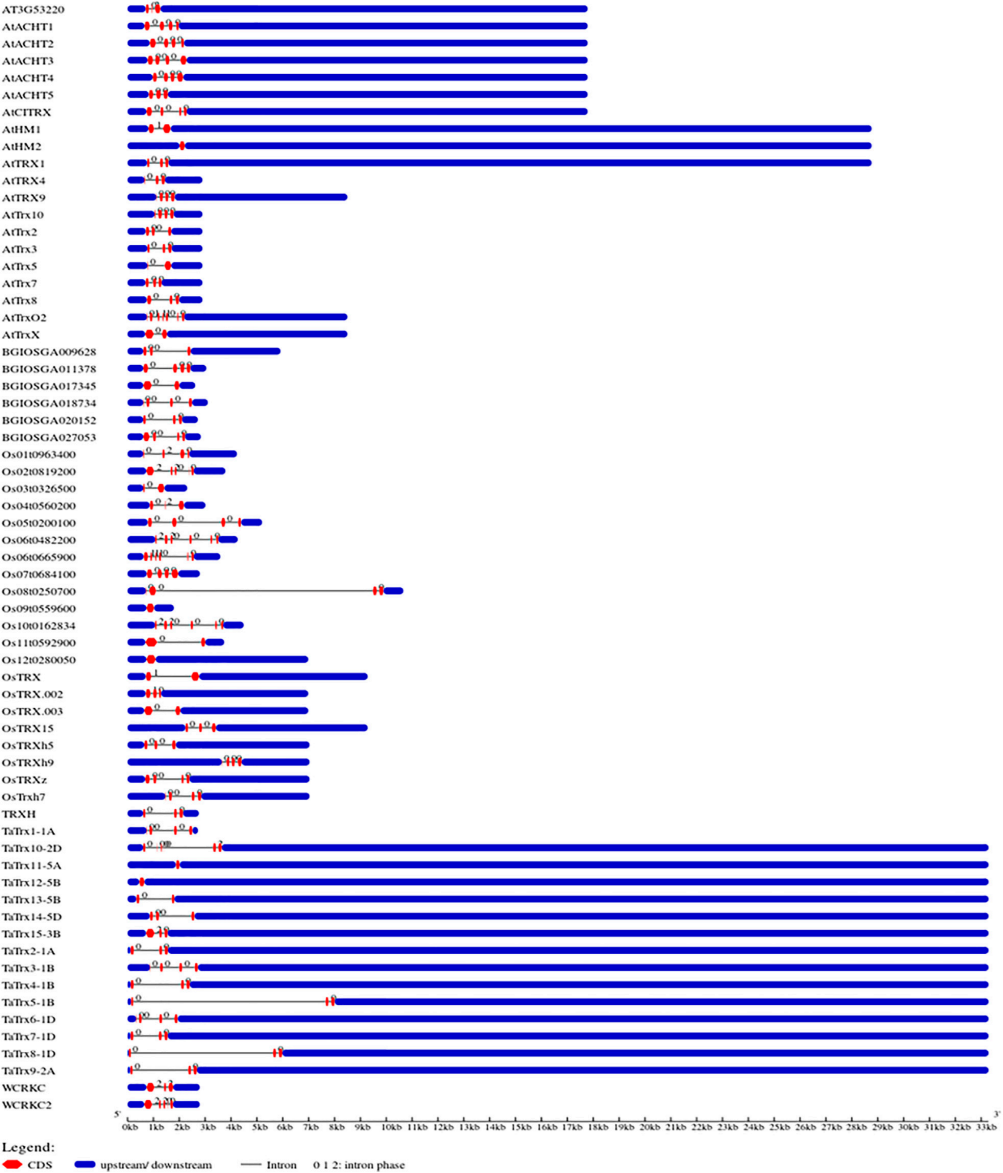
Illustration of gene structure of identified wheat TaTrx genes along with Trxs from *Arabidopsis* and rice, showing the distribution of exons/introns, and exon phase created in the Gene Structure Display Server (GSDS v2.0) (http://gsds.cbi.pku.edu.cn/).

In the present study, genomic sequences and cDNA sequences of 15 individual selected *TaTrx* genes were compared, and gene structure was obtained, as shown in Figure 3. Analysis of gene structure shows the presence of introns in all *TaTrx* genes, with the number of introns ranging from 0 to 5. *TaTrx11-5A* and *TaTrx12-5B* lack intron while *TaTrx10-2D* contains maximum of five introns. *TaTrx13-5B* possesses a single intron. *TaTrx3-1B* and *TaTrx6-1D* contain three introns, while the rest of the identified *TaTrx* genes contain two introns. *TaTrx5-1B* and *TaTrx8-1D* have the longest intron among all the identified *TaTrxs*, whereas all others have approximately similar lengths of introns.

**FIGURE 4:**
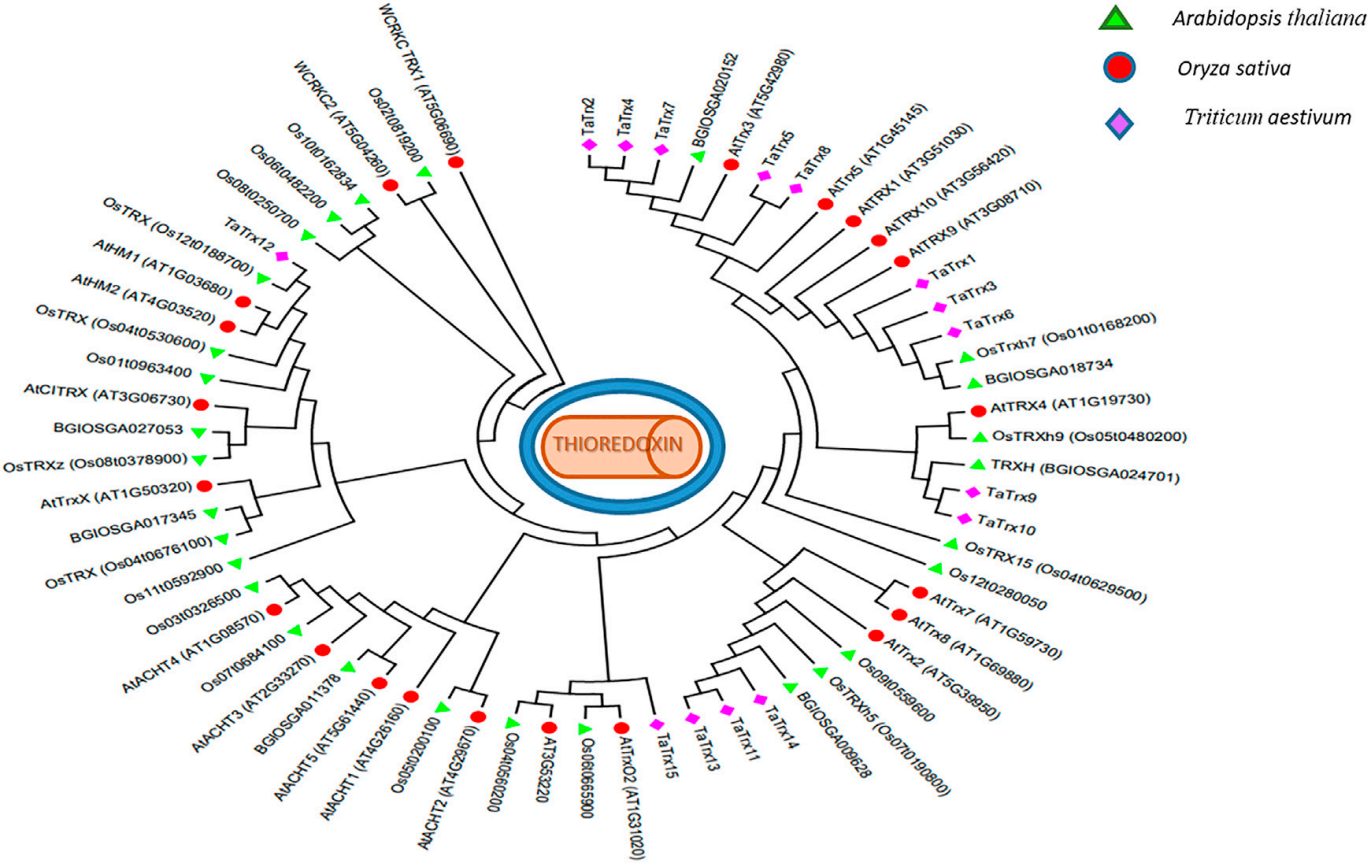
Phylogenetic tree constructed using MEGA X (v7) software (https://www.megasoftware.net/) of the identified wheat TaTrx protein along with *Arabidopsis* and rice Trxs.

Phylogeny analysis led to the clustering of a set of 65 protein sequences (Figure 4). The evolutionary relationship of identified 15 TaTrx proteins and Trx proteins in Rice (indica and japonica group) and *Arabidopsis* are represented by a phylogenetic tree constructed using the maximum likelihood method, suggesting Trx proteins mainly clustered into six major groups. Group I, the largest group, includes TaTrx2, TaTrx4, TaTrx5, TaTrx7, and TaTrx8 and two Trx from *Arabidopsis* one from rice. Group II contains TaTrx1, TaTrx3, and TaTrx6, three Arabidopsis, and two from Rice. TaTrx9 and TaTrx10 form the III group, TaTrx11, TaTrx13, and TaTrx14 form the IV group. Group V and Group VI contain only one Trx, TaTrx15, and TaTrx12. (Figure 4). All identified TaTrxs and Trx of *Arabidopsis* and rice shared a highly conserved Trx domain.

MEME software was used to analyze the conserved motifs of TaTrx proteins, and five distinct conserved regulatory motifs were predicted in *TaTrx* genes. All the five predicted regulatory motifs were conserved across all 15 TaTrx proteins schematically represented in Supplementary Figure S1. The sequences of conserved motifs with further information are provided in Supplementary Table S4.

### Putative Cis-Regulatory Element Identification in the Promoter Region of the *TaTrx* Genes

*TaTrx* cis-regulatory elements investigation revealed potential cis-acting regulatory elements (CAREs) responsive to various hormones and defense and stress-responsive elements. Hormone-related elements include MeJA-responsiveness (MeJARE), abscisic acid responsiveness (ABRE), and salicylic acid responsiveness (SARE). Some other detected elements were related to development responsiveness (GARE, ARE, Meristem expression) and abiotic stress (DRE, MYB, MYC, LTRE, LRE) (Supplementary Table S5).

Of the 15 *TaTrx* genes, nine contained elements related to defense and stress, while all *TaTrx* genes contained conserved cis-elements for various plant hormones. Among these 9 *TaTrx* genes, each *TaTrx1-1B*, *TaTrx3-1B*, *TaTrx5-1B*, *TaTrx6-1D*, *TaTrx11-5A*, *TaTrx15-3B* gene holding only 1, *TaTrx13-5B*, *TaTrx14-5D* possess 2, and *TaTrx8-1D* possesses 4 cis-acting elements involved in defense and stress responsiveness. All identified cis-acting elements involved in defense and stress responsiveness possess TC-rich repeats.

### Identification of miRNA Targets

Analysis of putative miRNA targets for *TaTrx* genes identified eight miRNAs that include targets for different *TaTrx* genes, as shown in Supplementary Table S6. The length of miRNA ranges from 19 nucleotides to 23 nucleotides. Supplementary Table S6 also includes information on the sequence of miRNA aligned with the target sequence. The mode of action of miRNA miR9673-5p, tae-miR9674a-5p, and tae-miR9676-5p is translational inhibition, while the rest of miRNA causes cleavage of mRNA. Among 15 *TaTrx* genes, *TaTrx*10-2D is putative target for tae-miR9674a-5p and tae-miR9658-3p miRNA. *TaTrx*2-1A gene is putative target for tae-miR396-5p and tae-miR9673-5p. Putative targets for *TaTrx*5-1B, *TaTrx*13-5B, *TaTrx*7-1D and *TaTrx*4-1B are tae-miR1136, tae-miR9666a-3p, tae-miR9674b-5p, and tae-miR9676-5p miRNA, respectively. TaTrx- miRNA interaction network model was constructed using Cytoscape that shows putative miRNAs for targeting *TaTrx* genes (Supplementary Figure S2).

### Physiochemical Properties of TaTrx Proteins

Physiochemical properties such as average residue weight (g/mol), charge, isoelectric point, molecular weight, theoretical PI, instability index, and grand average of hydropathicity (GRAVY) of 15 selected TaTrx proteins are listed in Table 2. Molecular weight (Mw) ranges from 12,858.97 g/mol (TaTrx5-1B) to 19,932.04 g/mol (TaTrx15-3B). Average residue weight ranges from 105.461 g/mol (TaTrx15-3B) to 111.319 g/mol (TaTrx14-5B), isoelectric point ranges from 4.8781 (TaTrx*2*-1A) to 8.3220 (TaTrx12-5B).

**TABLE 2:** Predicted physicochemical properties of TaTrx proteins.

S. no.	Protein	aa	Mol. weight g/mol	Theoretical PI	Instability index	Aliphatic index	GRAVY	Stability
1	TaTrx1-1A	131	14,506.60	5.15	24.09	78.09	−0.273	YES
2	TaTrx2-1A	130	13,750.04	5.12	17.02	94	0.377	YES
3	TaTrx3-1B	131	14,506.60	5.15	24.09	78.09	−0.273	YES
4	TaTrx4-1B	127	13,523.76	5.12	16.52	94.65	0.347	YES
5	TaTrx5-1B	188	12,858.97	5.2	32.7	90.08	0.064	YES
6	TaTrx6-1D	131	14,506.60	5.15	24.09	78.09	−0.273	YES
7	TaTrx7-1D	126	13,446.62	5.12	14.79	96.11	0.308	YES
8	TaTrx8-1D	119	12,977.22	5.06	31.09	102.44	0.224	YES
9	TaTrx9-2A	118	12,693.68	5.29	18.71	86.86	0.156	YES
10	TaTrx10–2D	136	14,963.27	5.32	13.89	90.37	0.065	YES
11	TaTrx11-5A	131	14,538.72	6.29	31.29	78.17	−0.101	YES
12	TaTrx12-5B	175	19,132.37	8.67	47.13	87.49	0.025	NO
13	TaTrx13-5B	131	14,582.77	5.9	30.84	77.4	−0.136	YES
14	TaTrx14-5D	131	14,556.74	5.9	29.36	78.17	−0.11	YES
15	TaTrx15-3B	189	19,932.04	8.46	45.72	80.53	−0.02	NO

Out of 15 Trx proteins, 13 are stable with an instability index of less than 40, while the remaining two proteins are unstable with index more than 40. The aliphatic index of identified Trx proteins ranges from 77.40 (TaTrx13-5B) to 102.44 (TaTrx8-1D). A set of seven (7) TaTrx proteins: TaTrx1-1A, TaTrx3-1B, TaTrx6-1D, TaTrx11-5A, TaTrx13-5B, TaTrx14-5B, and TaTrx15-3B showed the negative value of the grand average of hydropathicity (GRAVY) while the remaining eight (8) TaTrx proteins: TaTrx2-1A, TaTrx4-1B, TaTrx5-1B, TaTrx7-1D, TaTrx8-1D, TaTrx9-2A, TaTrx10-2D, and TaTrx12-5B were recorded with a positive value.

Prediction of subcellular localization of Trx proteins (Table 2; Supplementary Table S7) shows that one TaTrx is located each in chloroplast (TaTrx4-1B), chloroplast outer membrane (TaTrx15-3B), and in mitochondria (TaTrx12-5B), two TaTrx (TaTrx2-1A and TaTrx7-1D) are extracellular while that five TaTrx each are located in cytosol (TaTrx1-1A, TaTrx3-1B, TaTrx6-1D, TaTrx9-2A, TaTrx10-2D) and in the nucleus (TaTrx5-1B, TaTrx8-1D, TaTrx11-5A, TaTrx13-5B, TaTrx14-5D).

### Homology Modeling and 3D Structure Analysis of TaTrx Proteins

**FIGURE 5:**
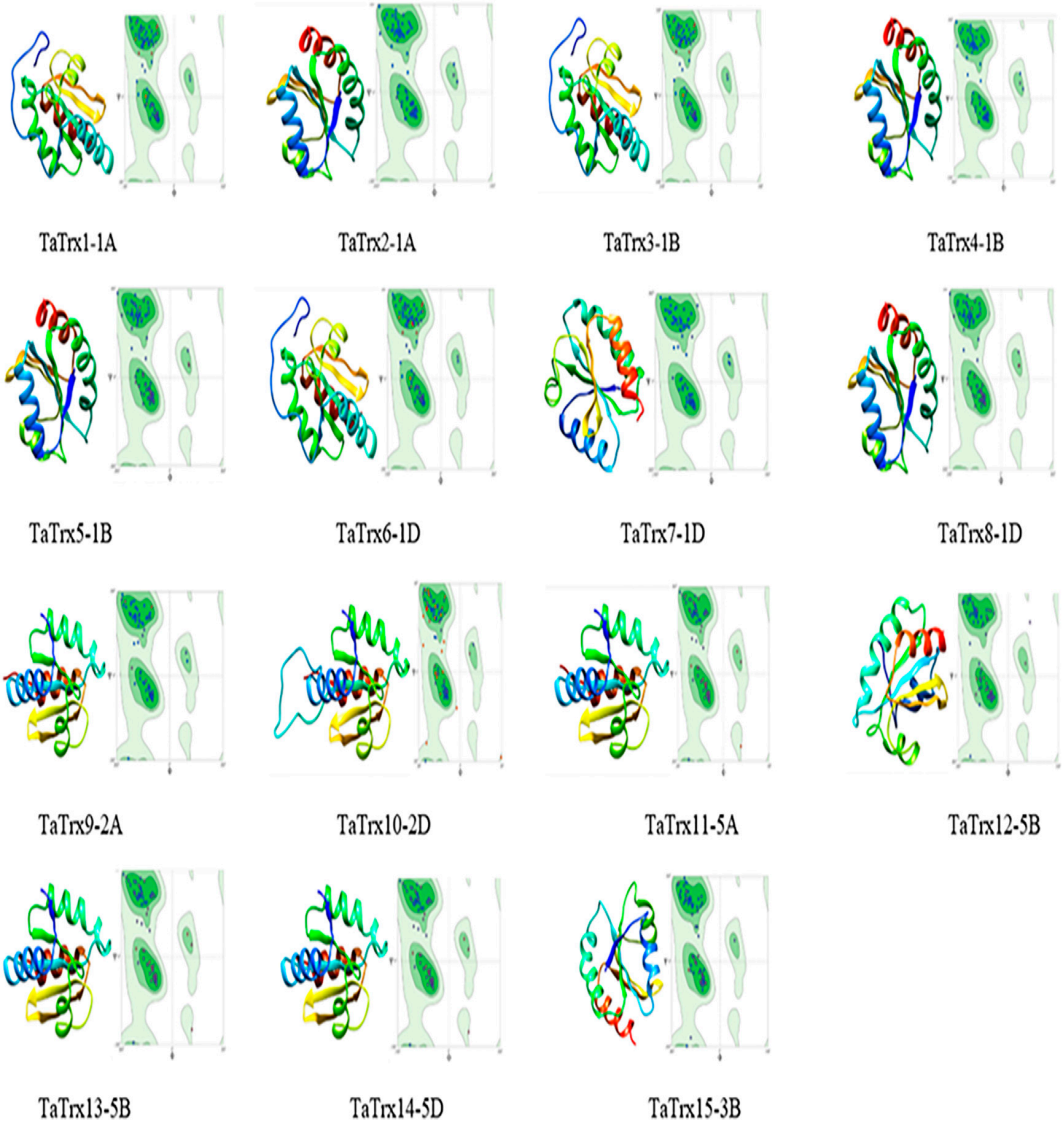
Automatic prediction of 3D structure and homology modeling of TaTrx proteins generated using UCSF chimera (http://www.cgl.ucsf.edu/chimera). Ramachandran plot of corresponding TaTrx protein showing most favored region and allowed region created using SWISS-MODEL server.

Three-dimensional (3D) structure of TaTrx proteins homology modeling (comparative modeling) is given in Supplementary Table S8, Figure 5. Homology modeling and 3D structure analysis showed that four TaTrx exist as dimers and 11 TaTrx as monomeric. TaTrx proteins were compared against templates 3d22.1.A, 2iwt.1.A, 2vlt.1.A, 2vm1.3.A, 1fb0.1.A, and 1faa.1.A from PDB, which unveils the structure of Poplar thioredoxin h, thioredoxin h2, thioredoxin h isoform 2, barley thioredoxin h isoform 1, spinach thioredoxin M, and spinach thioredoxin F, respectively.

The per cent sequence identity of the target TaTrx proteins with template protein, template protein ID, QMEAN, template description, oligo state, and Ramachandran favored per cent were shown in the Supplementary Table S8. The predicted 3D structure of TaTrx proteins is shown in Figure 5, along with the Ramachandran plot of the corresponding TaTrx protein. Ramachandran plot shows that more than 95% (in some proteins more than 99%) of the amino acid residues of thioredoxin proteins lies in the most favored area.

### Molecular Interaction Networks

The results of protein–protein interaction analysis done by STRING v11.0 for different TaTrx proteins are given in Supplementary Figures S3A–I. The PPI network shows interaction with thioredoxin reductases and several other uncharacterized proteins. Apart from thioredoxin reductase and a few uncharacterized proteins, TaTrx12-5B also interacted with chloroplastic peroxiredoxin. Protein–protein interaction analysis of TaTrx protein revealed interaction of Trx5 with NPR1 and NPR3 (Supplementary Figures S4, S5).

### Expression Analysis of Thioredoxin Genes in Wheat Seedlings

**FIGURE 6:**
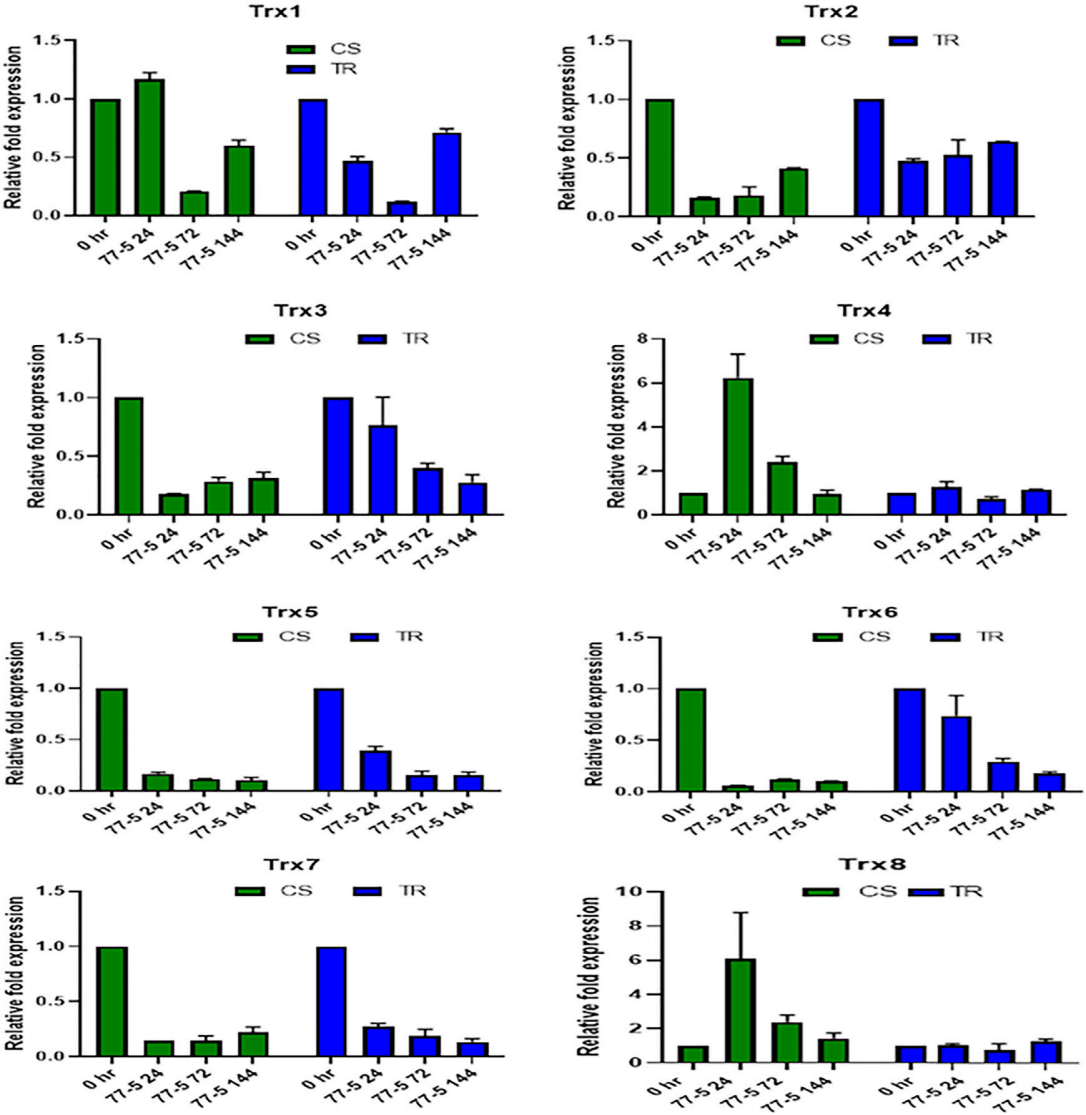
Expression analysis of Trx family genes (Trx 1, Trx 2, Trx 3, Trx 4, Trx 5, Trx 6, Trx 7, and Trx 8) in response to leaf rust inoculation. Chinese spring (CS) and introgression line of leaf rust resistance gene *(Lr9)* in CS, Transfer (TR). Leaf rust pathogen *Puccinia triticina* Eriks Pathotype 77-5 (121-R-63) uredospores were inoculated on 10 days old wheat seedlings and kept in a humidity chamber for 24 h. Leaf samples for qRT-PCR analysis were collected at 0 h after inoculation (HAI), 24 HAI, 72 HAI, and 144 HAI. Values are mean (±SE) of three biological replicates.

**FIGURE 7:**
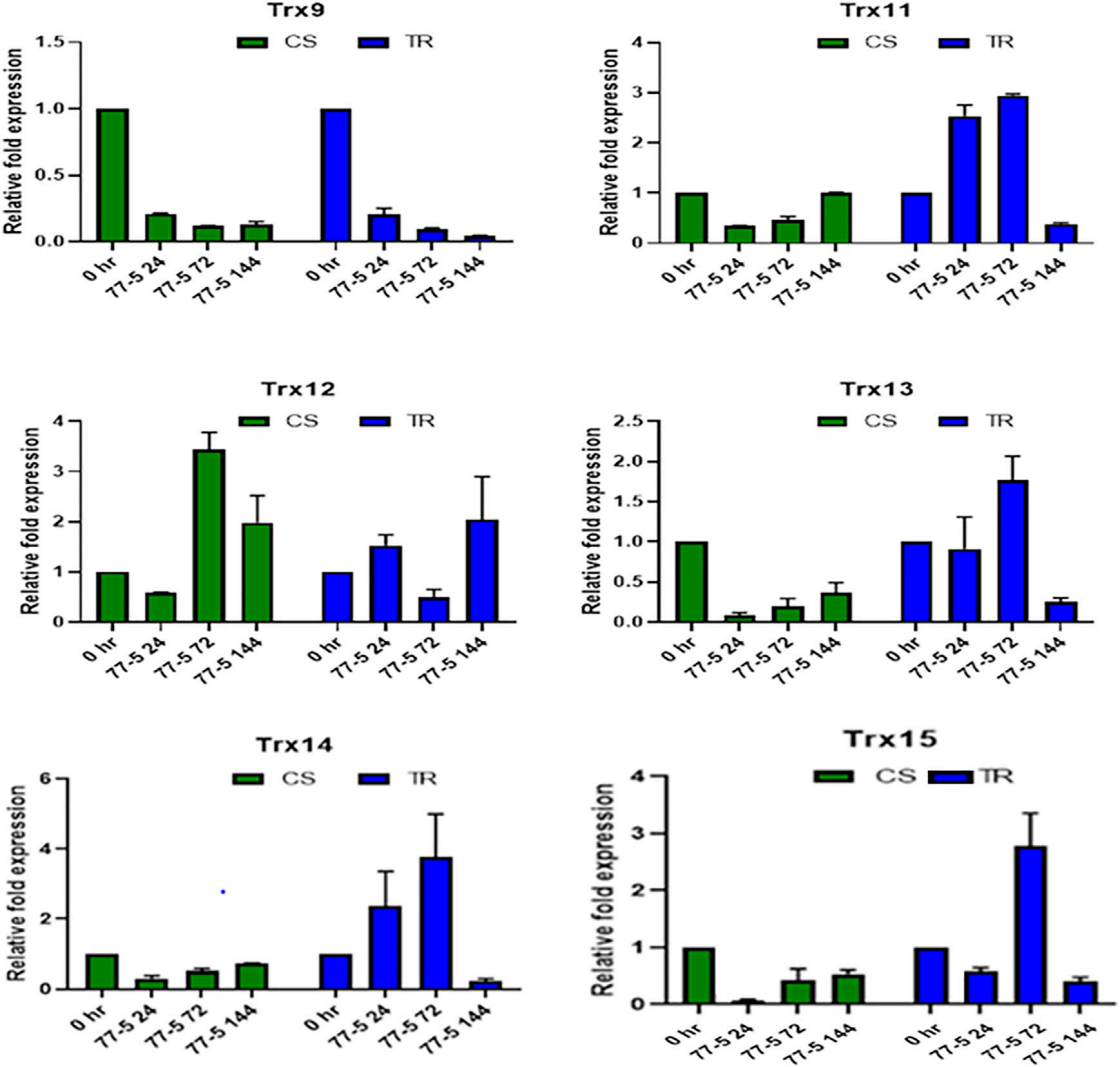
Expression analysis of Trx family genes (Trx 9, Trx 11, Trx 12, Trx 13, Trx 14, and Trx 15) in response to leaf rust inoculation. Chinese spring (CS) and Introgression line of leaf rust resistance gene *(Lr9)* in CS, Transfer (TR). Leaf rust pathogen *Puccinia triticina* Eriks Pathotype 77-5 (112-R-63) uredospores were inoculated on 10 days old wheat seedlings and kept in a humidity chamber for 24 h. Leaf samples for qRT-PCR analysis were collected at 0 hafter inoculation HAI, 24 HAI, 72 HAI, and 144 HAI. Values are mean (±SE) of three biological replicates.

The infection recorded a total fold change in the genes expression of selected TaTrx genes profiling during different time intervals (0 HAI, 24 HAI, 72 HAI, and 144 HAI). The expression level of selected *Trx* genes is depicted in Figures 6, 7. Among 15 *Trx* genes, the expression of *TaTrx4-1B* and *TaTrx8-1D* showed no or little change in expression in respect to control. *TaTrx3-1B*, *TaTrx5-1B, TaTrx6-1D*, *TaTrx7-1D*, and *TaTrx9-2A* showed a regular reduction in expression span of 24 HAI, 72 HAI, and 144 HAI in response to leaf rust infection after the inoculation in TR with respect to control. Whereas, *TaTrx11-5A* (3 fold, 72 HAI), *TaTrx13-5B* (3.36 fold, 72 HAI), *TaTrx14-5D* (3.86 fold, 72 HAI), and *TaTrx15-3B* (3 fold, 72 HAI) genes were significantly upregulated in expression in a span of 72 HAI and then declined sharply at 144 HAI (0.2 fold) in response to leaf rust infection in respect to control. Among the downregulated *TaTrx* genes, the expression of *TaTrx2-1A*, *TaTrx3-1B*, *TaTrx5-1B*, and *TaTrx6-1D* transcripts abundance was higher in resistance/incompatible interaction (TR) in comparison to susceptible/compatible interaction (CS) at different expression span.

### Localization and Accumulation of ROS (Superoxide Radical (SOR) and Hydrogen Peroxide) and Activity of Antioxidant Enzymes in Wheat Seedlings

**FIGURE 8:**
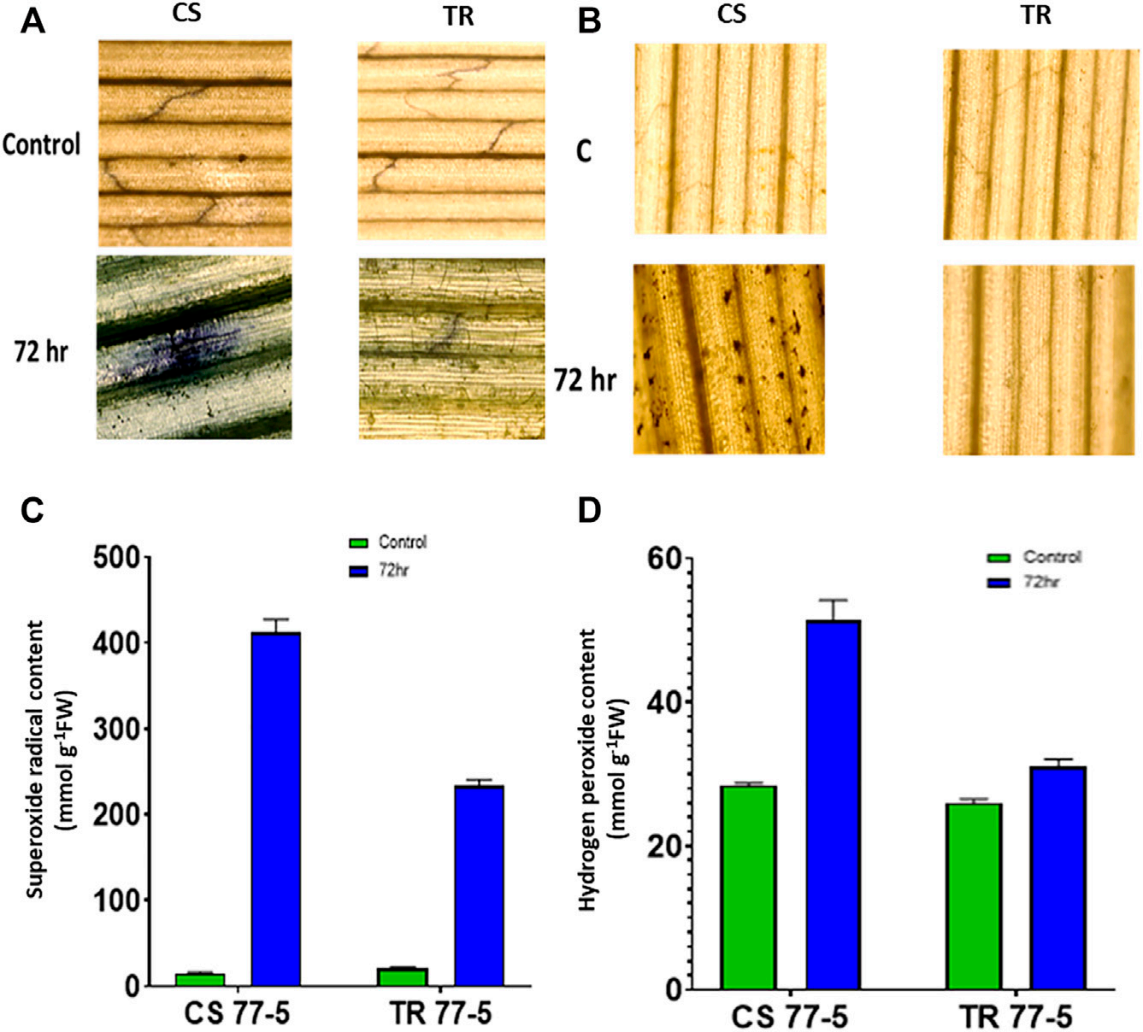
Effects of leaf rust pathogen on the localisation and quantification of reactive oxygen species, superoxide radical **(A,B)** and hydrogen peroxide **(C,D)**. Chinese spring (CS) and introgression line of leaf rust resistance gene *(Lr9)* in CS, Transfer (TR). Leaf rust pathogen *Puccinia triticina* Eriks Pathotype 77-5 (121-R-63) uredospores were inoculated on 10 days old wheat seedlings and kept in a humidity chamber for 24 h. Leaf samples for analysis were collected at 0 h after inoculation (HAI), and 72 HAI. Values are mean (±SE) of three biological replicates.

**FIGURE 9:**
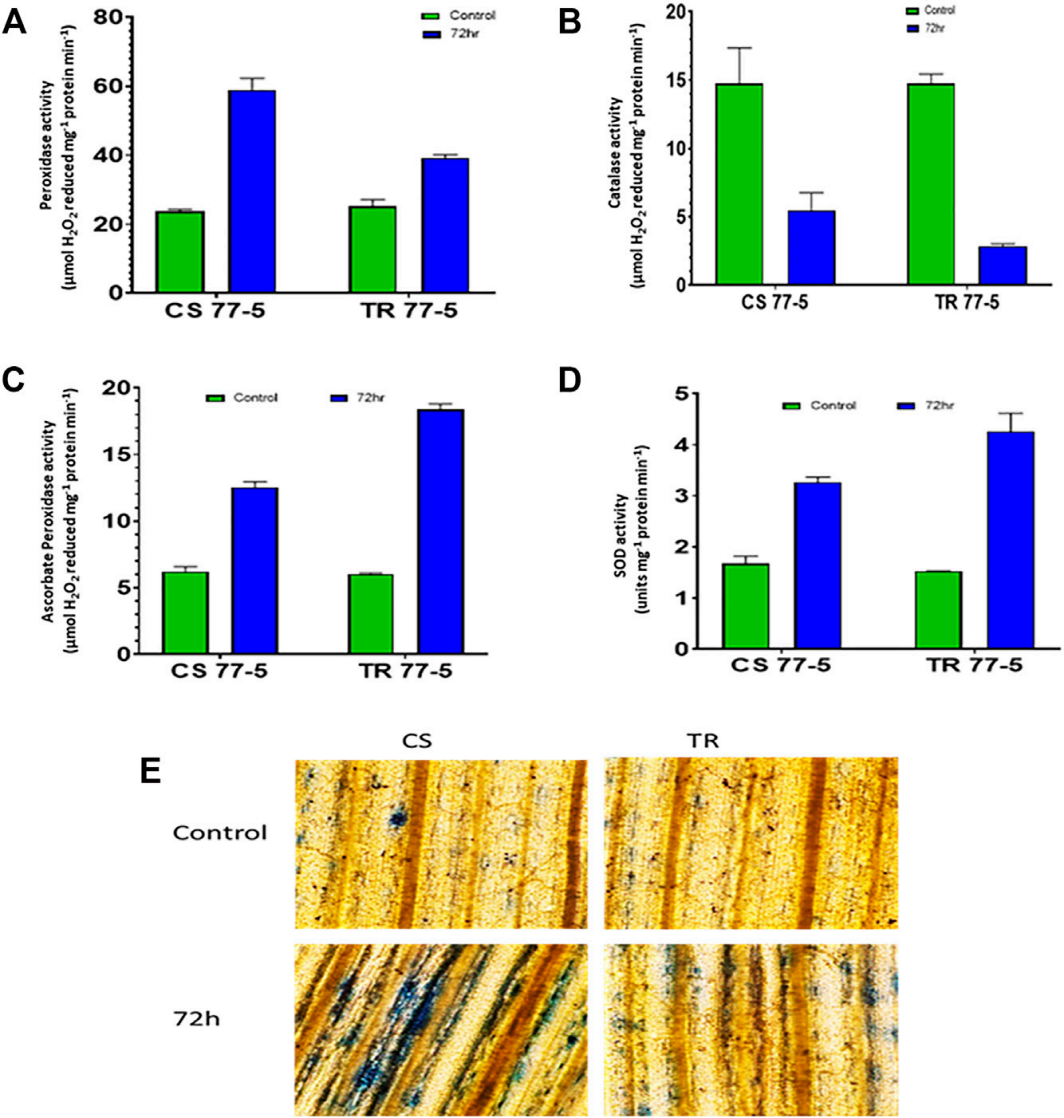
Effect of leaf rust pathogen on the activity of antioxidant enzymes **(A)** peroxidase, **(B)** catalase, **(C)** ascorbate peroxidase, **(D)** superoxide dismutase, and **(E)** membrane injury visualized by Evans blue staining of leaves of wheat genotypes Chinese spring (CS) and introgression line of leaf rust resistance gene (Lr9) in CS, Transfer (TR). Leaf rust pathogen *Puccinia triticina* Eriks pathotype 77-5 (121-R-63) uredospores were inoculated on 10-day-old wheat seedlings and kept in a humidity chamber for 24 h. Leaf samples for analysis were collected at 0 h after inoculation (HAI) and 72 HAI. Values are means (±SE) of three biological replicates.

Compatible interaction of leaf rust resulted in ROS burst as indicated by localization and content of SOR and hydrogen peroxide. As presented in Figures 8A,C, the occurrence of dark blue color and the appearance of the brown-colored product confirmed the presence of SOR and hydrogen peroxide. Spectrophotometric assay of SOR and hydrogen peroxide also showed higher ROS levels in the compatible interaction of leaf rust (Figures 8B,D). Leaf rust inoculation triggered the activity of antioxidant enzymes Peroxidase (POX), Superoxide dismutase (SOD), and Ascorbate peroxidase (APX), while catalase activity was reduced after inoculation with the pathogen. The magnitude of upregulation in SOD and APX activity was higher (approximately three-fold) in incompatible interaction as compared to compatible interaction (approximately two-fold) that could have arrested the ROS burst as depicted as a lower abundance of both the ROS considered in the study (Figure 9).

### Analysis of Variance

Analysis of variance (ANOVA) showed that out of 15 identified TaTrx, 13 TaTrx were significant at the expression level compared to control. At the same time, the remaining two genes, *TaTrx4-1B* and *TaTrx8-1D,* were non-significant (Supplementary Table S9). A heat map showing the Pearson correlation matrix relationship between expression of TRX genes, ROS accumulation and antioxidant enzyme activities in leaves of two contrasting wheat genotypes, CS and TR is given in Supplementary Figure S6. The correlation heat map showed that the accumulation of APX, SOD, POX, SOR, and HP (H_2_O_2_) positively correlated with the *TaTrx* genes exhibiting upregulation against infection. On the other hand, the accumulation of CAT showed a positive correlation with the *TaTrx* genes showed downregulation during the span of infection (Supplementary Figure S6).

## Discussion

Thioredoxin is a crucial factor that plays an essential role in host resistance and several other plant constituents. ROS production and scavenging are associated with the modulation of plant immune signaling pathways (Vieira Dos Santos and Rey, 2006). Trx acts as a critical regulator element in defense mechanisms and is essential for modulating the redox status of components of defense pathways. The study was based on genome-wide analysis of *Thioredoxin* genes in bread wheat that revealed one set of 15 *TaTrx* genes based on *in silico* expression analysis for biotic stress responses of wheat. Genome-wide analyses of *Thioredoxin* genes have also been performed in *Arabidopsis* and rice (Meyer et al., 2008; Nuruzzaman et al., 2012). Several genes included the Trx domain in various crop plants such as 74 genes in Arabidopsis, 61 in Rice, 11 in sorghum, and 36 in maize (Nuruzzaman et al., 2012).

A comparative analysis with the conserved or diverse *Trx* genes among the different wheat species; *T. Urartu* (A genome), *T. turgidum* (AB genome), and *A. tauschii* (D Genome), led to the identification of conserved genes on different three genomes A, B, and D. Both *T. aestivum* and progenitor’s species shared a similar maximum number of genes on three different genomes, i.e., genome A with ∼10 Trx genes, B carried ∼21 Trx genes, and Genome D contributed a set of ∼10 Trx genes. This is evidence that the Trx gene is conserved in the wheat genome throughout evolution.

Out of 15 TaTrxs, *TaTrx15-3B* (3 fold at 72 HAI) putatively localized in chloroplast was found to be upregulated in incompatible interaction with leaf rust pathogen. Most chloroplastic Trxs are involved in the photosynthetic process and respond to stress and hormone signaling (Fernández-Trijueque et al., 2019). Overexpression of a chloroplastic thioredoxin NtTRXh3 in tobacco showed to be involved in enhanced resistance against cucumber mosaic virus (CMV) and tobacco mosaic virus (TMV) (Sun et al., 2010). This study showed that of 15, 5 TaTrx genes (TaTrx1-1A, TaTrx3-1B, TaTrx6-1D, TaTrx9-2A, and TaTrx10-2D) located in cytoplasm either showed no or little change in expression with respect to treatments; however, the previous studies reported that in most of the cases cytosolic Trxs such as AtTrxh5 against *Pseudomonas syringae* and *Cochliobolus victoriae*, CITRX in response to *C. fulvum* were upregulated and involved in defense responses. Based on the location of three highly upregulated TaTrx genes, we found that nuclear Trxs were upregulated in response to leaf rust likely function in plant immunity.

In this study, we predicted the physiochemical properties, including theoretical PI, molecular weight, instability index, aliphatic index, and grand average of hydropathicity (GRAVY) of TaTrx proteins. The p^H^-related characteristics of a protein depend upon theoretical PI; at this point, protein has no charge and is less soluble, which facilitates the isolation of protein (Righetti, 2004; Audain et al., 2016). *In vivo* stability of a protein is inferred from the instability index calculated by the dipeptide composition-based method; a value less than 40 signifies that protein is stable, while a value more than 40 means that protein is unstable (Gamage et al., 2019). In the study, 13 proteins have an instability index value of less than 40, while two have more than 40, indicating the stable nature of the TaTrx protein. The aliphatic index (AI) is the relative volume occupied by side aliphatic amino acid residues that signify the thermostability of a protein (Ikai, 1980). All selected TaTrx proteins in wheat have a wide range of the aliphatic index. This suggests the wide range of thermostability of the Trx protein. In the present study, TaTrx proteins showed both negative (7 proteins) and positive (8 proteins) GRAVY values. GRAVY measures solubility and hydrophobicity or hydrophilicity; negative value specifies hydrophobic nature while a positive value specifies the hydrophilic nature of a protein (Fernández-Fernández and Corpas, 2016).

Phylogeny analysis of *TaTrx* and *Trx* of *Arabidopsis* and rice indicated that these belong to six different groups having similarities in exon-intron position and presence of domain. Genomic and CDS sequences were retrieved and used to analyze gene structure, indicating that the number of exons ranges from 1 to 6 while introns range from 0 to 5. Highly upregulated *TaTrx* genes (*TaTrx*11, *TaTrx* 13, *TaTrx*14, and *TaTrx*15) have 0–2 introns and are shorter than other identified Trx proteins. It is well known that introns play a key regulator role in alternative splicing (AS) and non-sense-mediated decay (NMD) (Kalyna et al., 2012), and it has been reported that genes with the fewer number of introns allow plants to respond more quickly against stress (Jaffares et al., 2008; Zheng et al., 2020). Therefore, identified *TaTrx* genes having fewer introns may express highly and quickly against leaf rust pathogen. However, the role of intron number on the function of TaTrx proteins needs further validation. Conserved motif analysis revealed that Trx contains five conserved regulatory motifs. All other Trx proteins, except four highly upregulated TaTrxs, have a similar pattern of conserved motifs, representing their involvement in gene regulation. Out of four highly expressed *TaTrxs*, *TaTrx*15-3B possesses only one motif identical to the other three upregulated Trx proteins. We can predict that the dissimilarity of TaTrx15 protein from others may provide its specificity during resistance. The *TaTrx15-3B* gene is located in chloroplast, while the remaining three highly upregulated genes are present in the nucleus showed significant upregulation during infection at different cellular compartment levels.

In the present study, homology modeling and 3D structure analysis showed that proteins shared more than 95% (in some proteins more than 99%) similarity. According to the PROCHECK algorithm, a quality protein model is expected to possess more than 90% amino acid residues in the most favored region (Laskowski et al., 1993), which advocates that our predicted models are of good quality. In our study, promoter regions of *TaTrx* genes possess CAREs as follows: MeJARE, ABRE, MYB, G-box, and W-Box. G-box and ABRE provide the suitable binding site for bZIP TFs that regulates and plays a vital role in NPR1 (NONEXPRESSOR OF PATHOGENESIS-RELATED GENES 1) mediated SAR (Heinekamp et al., 2004). Cis-acting regulatory elements are essential in response to biotic and abiotic stress (Sheshadri et al., 2016). Jasmonic acid and salicylic acid plays a vital role in orchestring signaling for SAR (Fu et al., 2012; Heil et al., 2012). As the SA level increases after pathogen infection during basal resistance, SA binds to NPR4 and releases more NPR1, which activates the SAR (Fu et al., 2012). MeJA act as an activator of the antioxidant system in plants during the production of the damaging level of ROS (Yu et al., 2018). Along with Trxs, MeJA may play a crucial role in ROS homeostasis during pathogen infection. Also, the protein–protein interaction showed Trx is a putative partner in interaction with NPR1.

Along with regulatory elements, some miRNAs were also predicted to be associated with the modulation of gene expression. Eight putative miRNAs were discovered that seven different target *TaTrx* genes. Differential expression of miRNAs has been observed during defense responses (Ramachandran et al., 2020). Identified *Trx* gene, *TaTrx13-5B* (upregulated during leaf rust infection) is a putative target for tae-miR9666a miRNA while the Trx gene *TaTrx5-1B* gene (downregulation during infection) is a putative target of MIR1136. The expression of miRNA tae-miR9666a and MIR1136 was found to be downregulated and upregulated, respectively, during treatment in stripe rust infected spring wheat cultivar (Ramachandran et al., 2020). This supports our study that downregulation of tae-miR9666a during the resistance to leaf rust might be involved in the upregulation of the expression of *TaTrx13-5B* in wheat. In contrast, the change in the expression level of MIR1136 could be associated with the downregulation of its target *TaTrx5-1B* gene.

Although most of the proteins in wheat are still uncharacterized, protein–protein interaction analysis using STRING recognizes that all TaTrxs interact with thioredoxin reductase. However, protein–protein interaction analysis in *Arabidopsis* revealed Trx5, thioredoxin H-type 5 (cytosolic thioredoxin) acts as a reducer of disulphide bridges and interacts with NPR1, NPR3 involved in systemic acquired resistance (SAR) and other thioredoxins (Supplementary Figures S4, S5). From the previous and present studies, it is clear that Trxs have a diverse role in plant immunity. Recently, a study provided insights into wheat’s molecular interaction network during the defense response pathway against stripe rust (*P. striiformis* f.sp. *tritici*). A thioredoxin gene has been identified and cloned in wheat (*TaTrxh1)* showed interaction with *TaCP1* (RD-19 like Cysteine protease) that enhances resistance against wheat stripe rust (*P. striiformis f.* sp. *tritici*) through modulation of PCD *via* SA dependent defense signaling pathway. Overexpression of *TaTrxh1* induces cell death, and *TaTrxh1* expression is induced significantly in response to *P. striiformis f.* sp. *tritici* infection (Shi et al., 2021). *TaTrx13-5B* (3.36 fold) showed upregulation in response to leaf rust infection with maximum expression at 72 HAI. This result aligned well with the data obtained in the *in silico* analysis experiment regarding stripe rust and powdery mildew disease (Figures 1, 7), as the expression of *TaTrx13-5B* transcript was also upregulated under both the biotic stresses. Therefore, it indicates that *TaTrx13-5B* could be the Trx involved in response to most biotrophic pathogens.

Superoxide production, a ROS, was first reported to be associated with potato’s hypersensitive response (HR) to infection of an incompatible race of *Phytophthora infestans*. Out of three inoculation spans (24 HAI, 72HAI, and 144HAI), maximum accumulation of superoxide and SOD and CAT activity was observed at 72 HAI. Localization of superoxide was observed in the case of the incompatible one but not in the compatible one (Doke, 1983). Similar results were observed from our experiment of superoxide localization in response to leaf rust that showed maximum accumulation at 72 HAI. In barley, H_2_O_2_ accumulation started at 6 HAI in response to powdery mildew infection and increased with time, HR was observed during 24 HAI (Thordal-Christensen et al., 1997). In our case, maximum accumulation of H_2_O_2_ was observed during 72 HAI (Figure 8). In uninoculated lettuce plants, H_2_O_2_ was localized in secondary thickening of xylem vessels while inoculation with *Pseudomonas syringae pv phaseolicola* causes highly localized accumulation of H_2_O_2_ resulting in HR (Bestwick et al., 1997). We found a similar accumulation of H_2_O_2_ around the leaf rust spores (Figure 8). Study of root apoplast secretome in wilt resistance *Gossypium barbadense* in response to *Verticillium dahliae* showed upregulation of ROS and defense-related proteins. Silencing a thioredoxin GbNRX1 showed that Trx is associated with ROS metabolism and is required for resistance against *Verticillium dahliae* in cotton (Li et al., 2016). Three types of Trx-m and a Trx-x in *Arabidopsis thaliana* were found to be upregulated in response to H_2_O_2_ accumulation and induce H_2_O_2_ tolerance (Issakidis-Bourguet et al., 2008). Thioredoxin, *PsTrx-h1* in pea was reported to be significantly induced in response to oxidative stress and showed that PsTrx-h1 was involved in redox homeostasis (Traverso et al., 2007). A recent study in wheat clearly showed that silencing of a wheat thioredoxin *TaTrx1* reduces the level of accumulation of ROS in response to *P. striiformis* f. sp. *tritici* infection.

**FIGURE 10:**
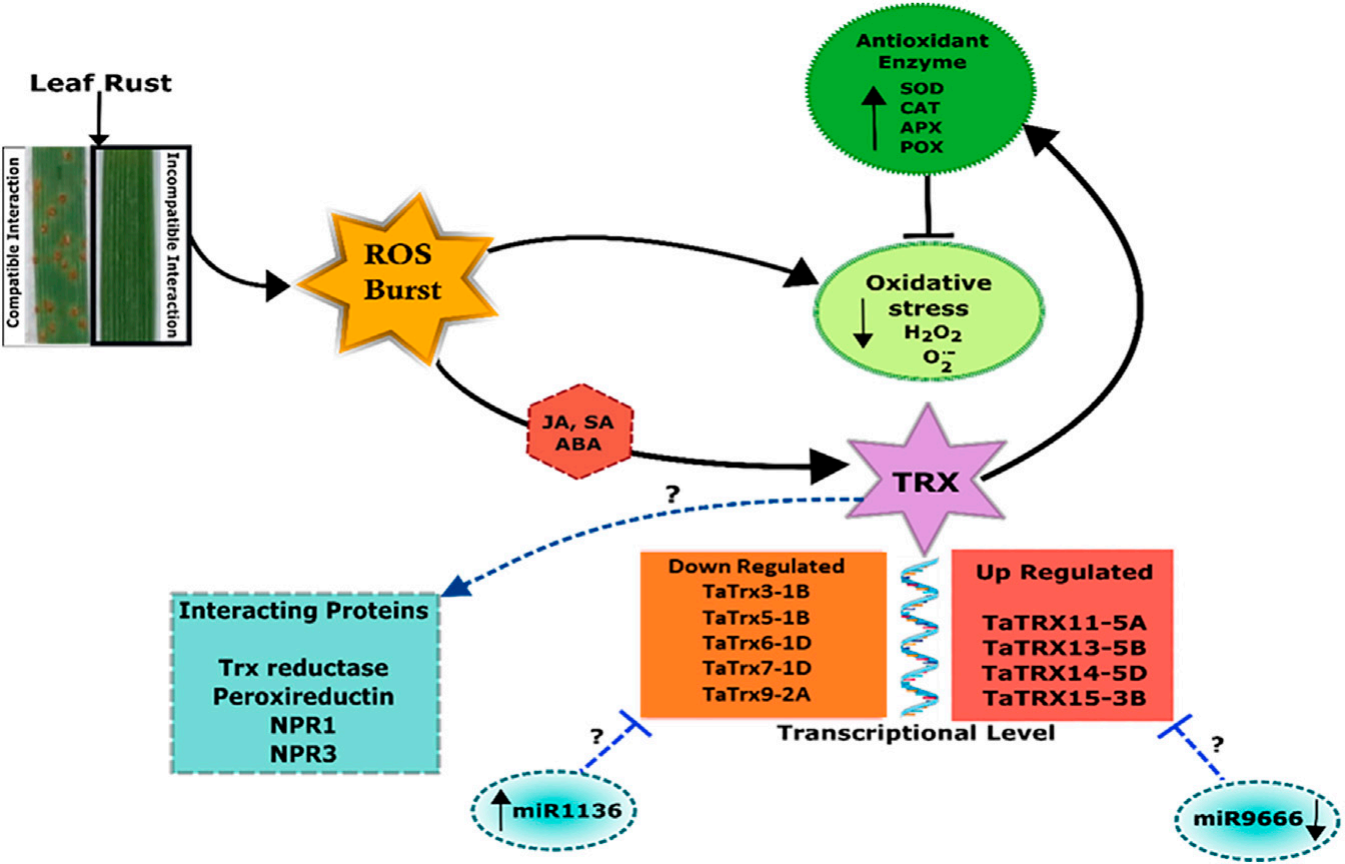
Schematic model showing thioredoxin (Trx)-mediated regulation of incompatible interaction between wheat and leaf rust pathogen. Figure is made based on the results and *in silico* prediction (enclosed in dashed lines).

Further, estimation of H_2_O_2_ in TaTrx1 silenced plants following inoculation with *P. striiformis* f. sp. *Tritici* race CYR-23 showed a significantly reduced area of H_2_O_2_ production at 120 HAI as compared to control plants. Parallel to this, the expression of antioxidants TaCAT and TaSOD were significantly induced as expected (Shi et al., 2021), which supports our study of the involvement of TaTrx in ROS homeostasis and ROS-dependent defense. We found that compatible interaction of leaf rust resulted in ROS burst as indicated by localization and content of SOR and hydrogen peroxide. Leaf rust inoculation triggered the activity of antioxidant enzymes peroxidase (POX), superoxide dismutase (SOD), and ascorbate peroxidase (APX), while catalase activity was reduced after inoculation with the pathogen (Figure 9). The magnitude of upregulation in SOD and APX activity was higher in incompatible interaction, which could have arrested the ROS abundance. Based on our results, we can say that Trxs in wheat also act as an antioxidant and are involved in the scavenging of ROS and reduce oxidative stress during incompatible interaction with leaf rust. Based on superoxide and H_2_O_2_ quantification and localization, it is inferred that ROS and antioxidant enzymes attained a maximum level at 72 HAI. Upregulated *Trx* genes also showed maximum expression during 72 HAI. The correlation of *Trx* gene expression, accumulation of ROS, and antioxidant enzymes at 72 HAI also indicates a significant association. For example, as depicted in the correlation heat map (Supplementary Figure S6), *TaTrx1-1A* and *TaTrx4-1B* expression showed a positive correlation with catalase activity and negative correlation with ROS accumulation *TaTrx2-1A* and *TaTrx6-1D* showed a positive correlation with most of the antioxidant enzymes. The perturbation in ROS homeostasis and gene expression indicates the involvement of thioredoxins (which has a regulatory role as antioxidant enzyme activity) in ROS homeostasis and leaf rust resistance in wheat (Figure 10).

## Conclusion

The wheat genome contains 42 members of the *Trx* gene family unevenly distributed on the seven chromosomes of A, B, and D genomes. Expression profiles of selected 15 Trxs, estimation, and localization of ROS demonstrate that the *Trx* gene family is associated with ROS homeostasis during biotic stress. Although previous studies indicated the participation of Trxs in various metabolic processes, their role in plant immunity is so far not entirely understood. Overall, this study provides insights into the function of the *Trx* gene family in response to leaf rust.

## Data Availability

The original contributions presented in the study are included in the article/Supplementary Material, further inquiries can be directed to the corresponding authors.
