# Supplementary figures and images for: Genome-Wide Identification and Expression Analysis of the Thioredoxin (Trx) Gene Family Reveals Its Role in Leaf Rust Resistance in Wheat (Triticum aestivum L.)
Source: Front Genet. 2022 Mar 25;13:836030. doi: 10.3389/fgene.2022.836030 (PMC8990325; doi:10.3389/fgene.2022.836030)

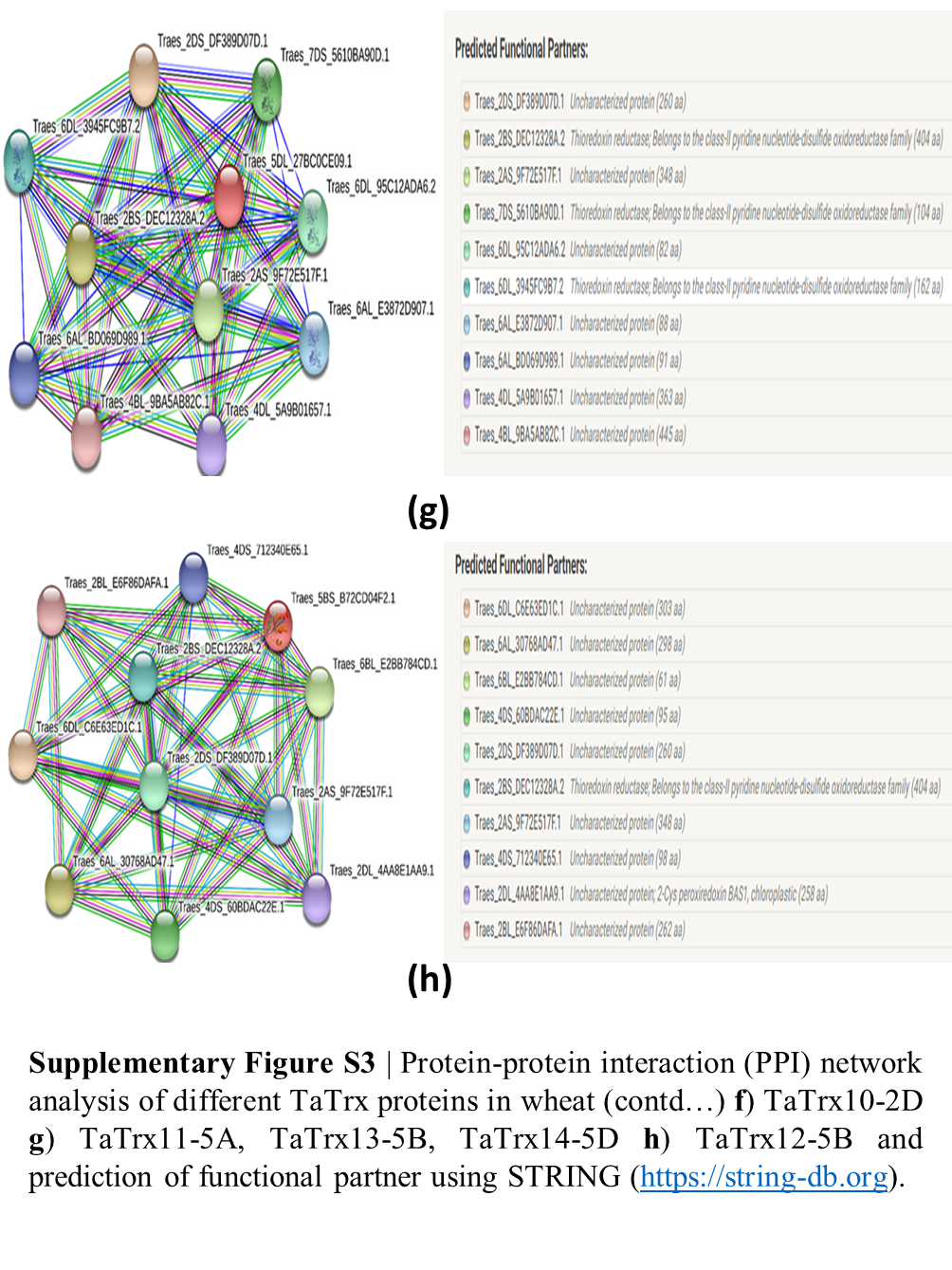

Supplement: Supplementary file 1 [file Image6.TIF]

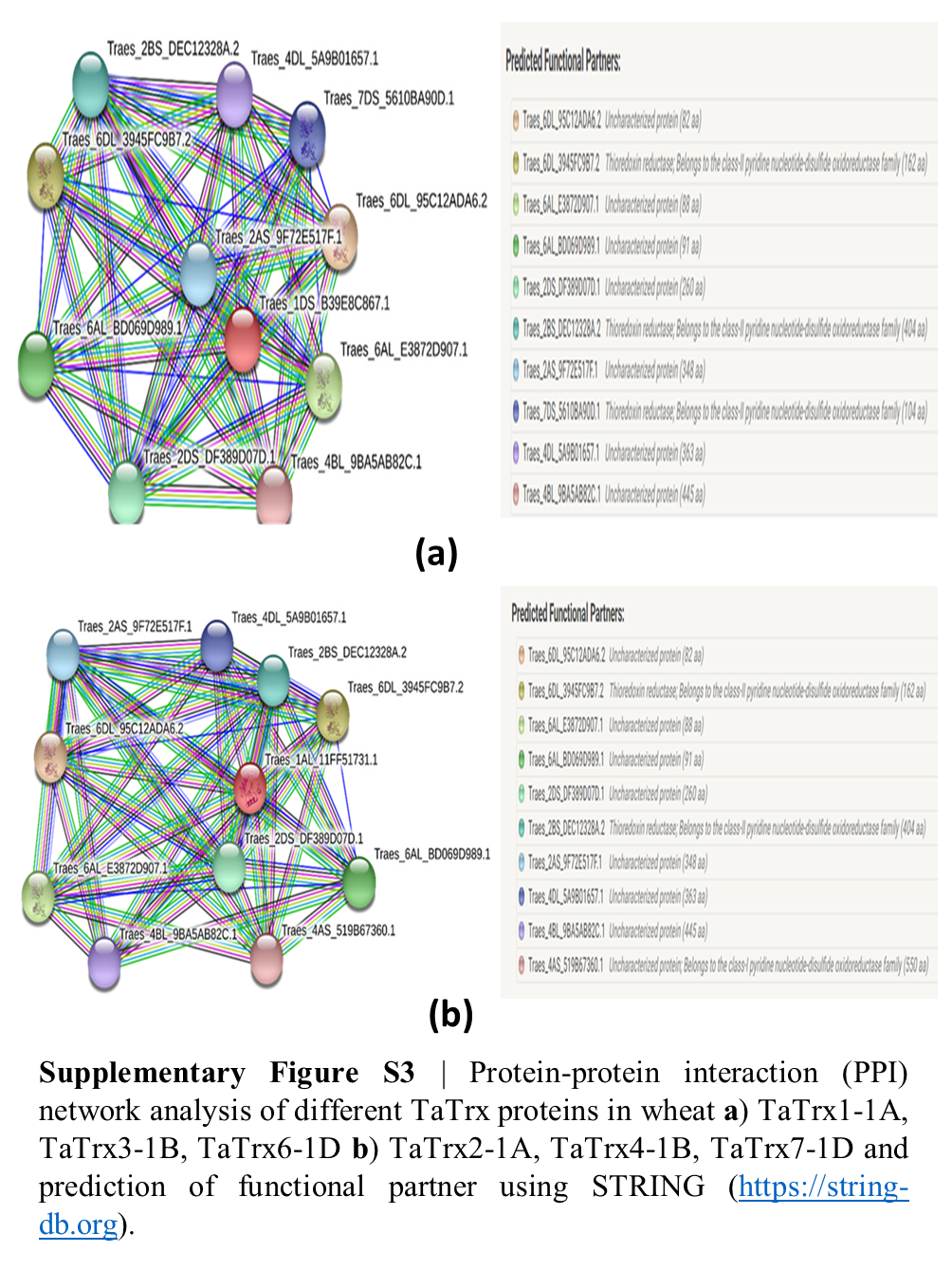

Supplement: Supplementary file 2 [file Image3.TIF]

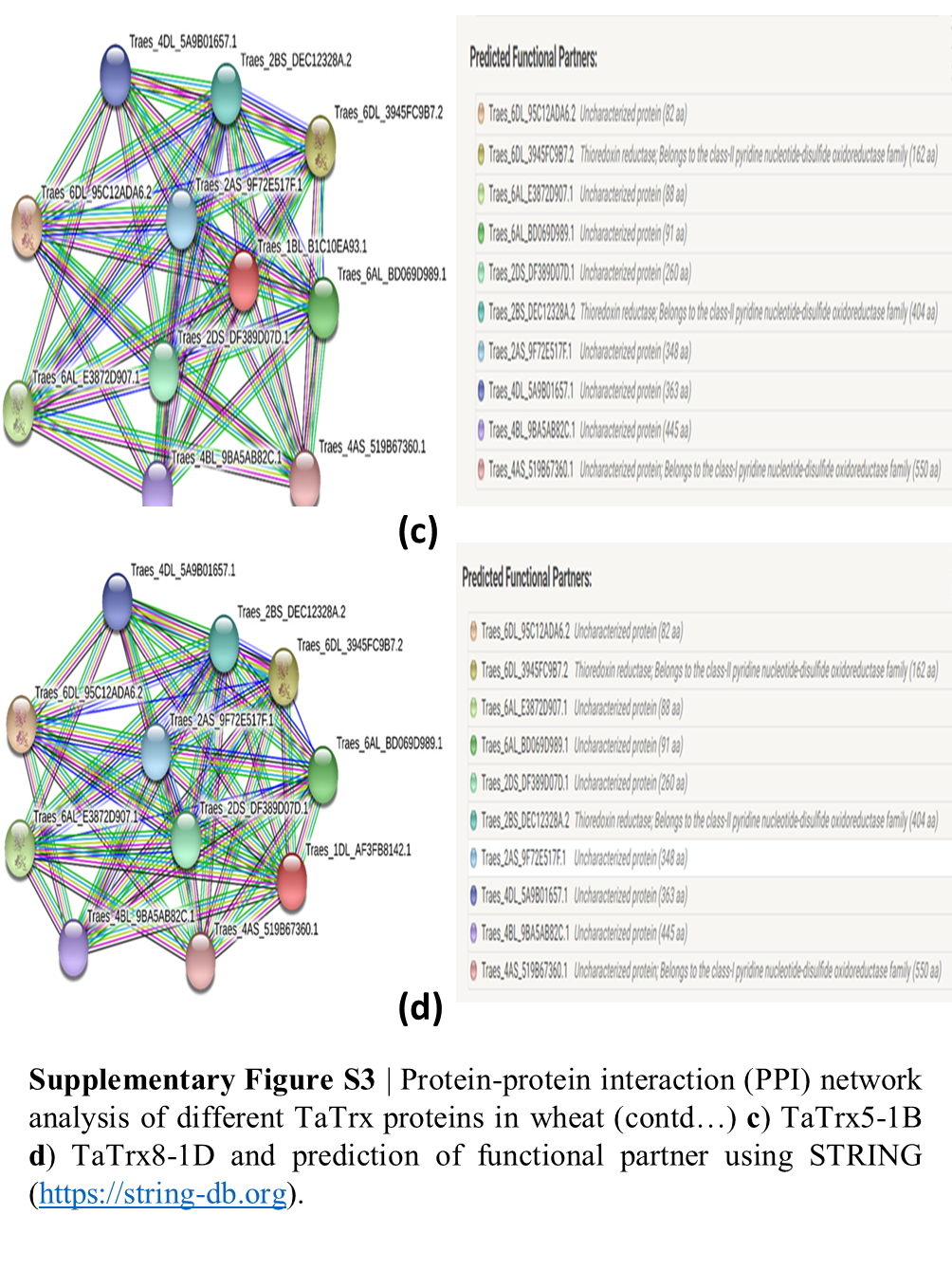

Supplement: Supplementary file 3 [file Image4.TIF]

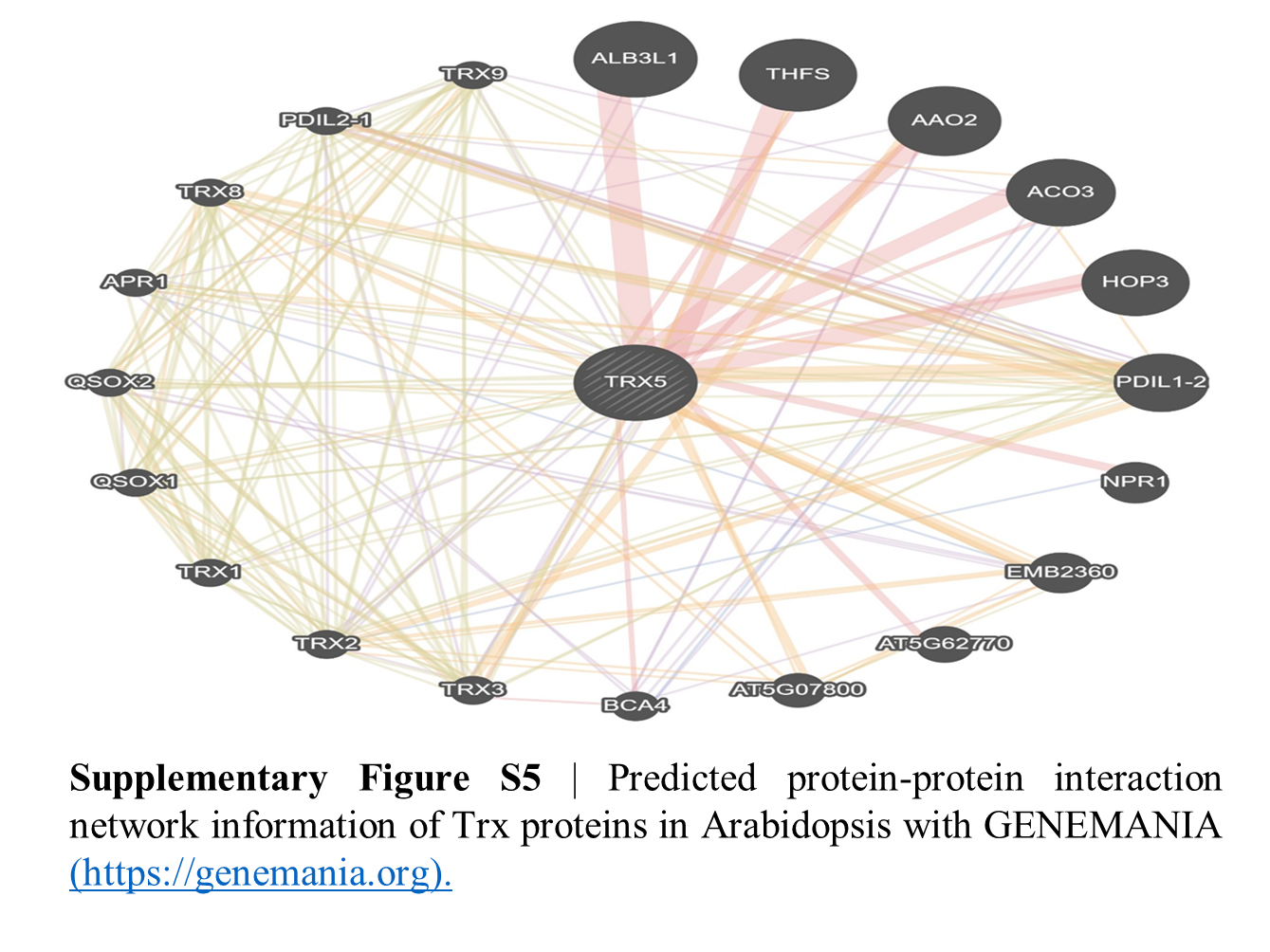

Supplement: Supplementary file 4 [file Image9.TIF]

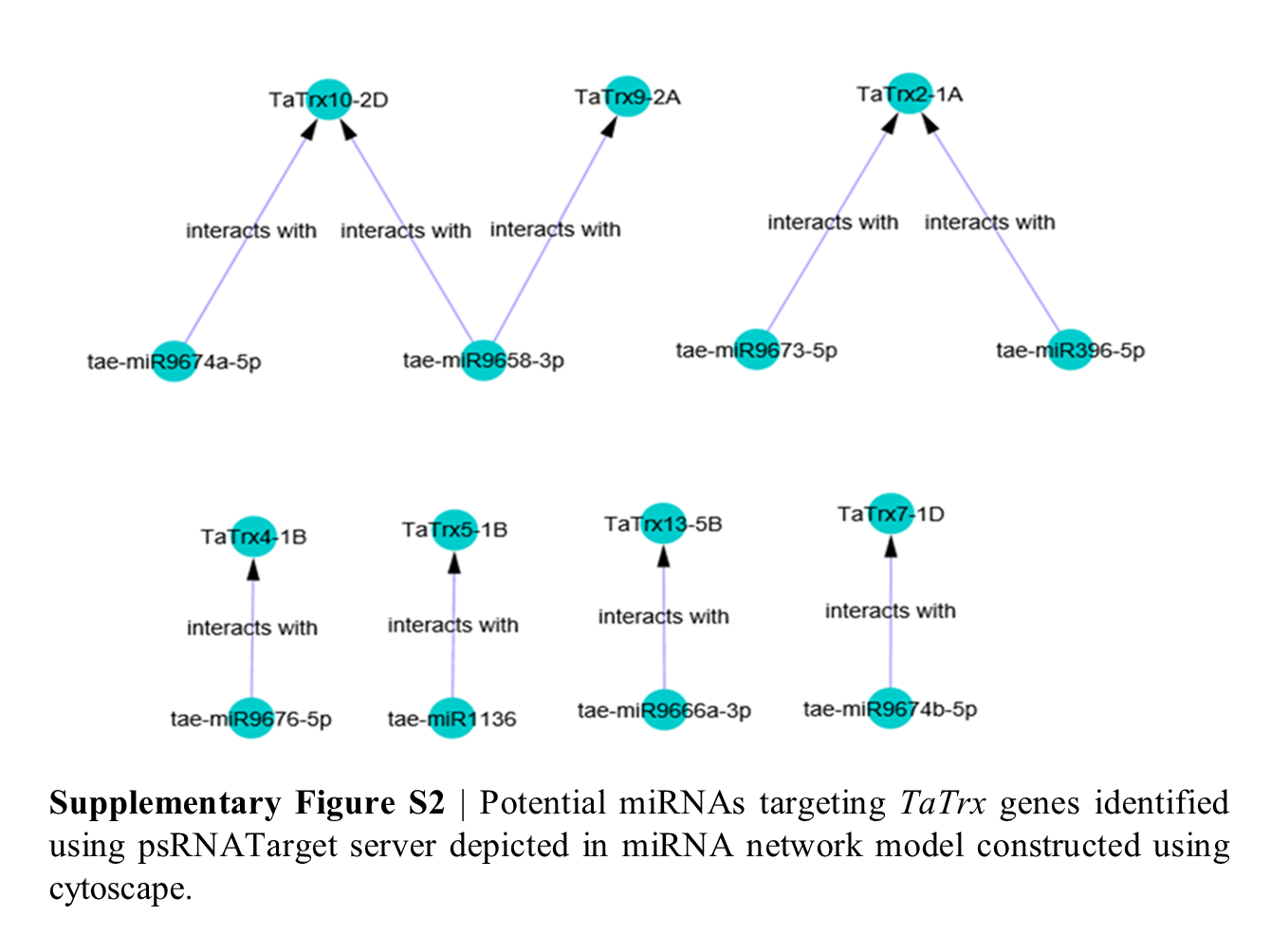

Supplement: Supplementary file 5 [file Image2.TIF]

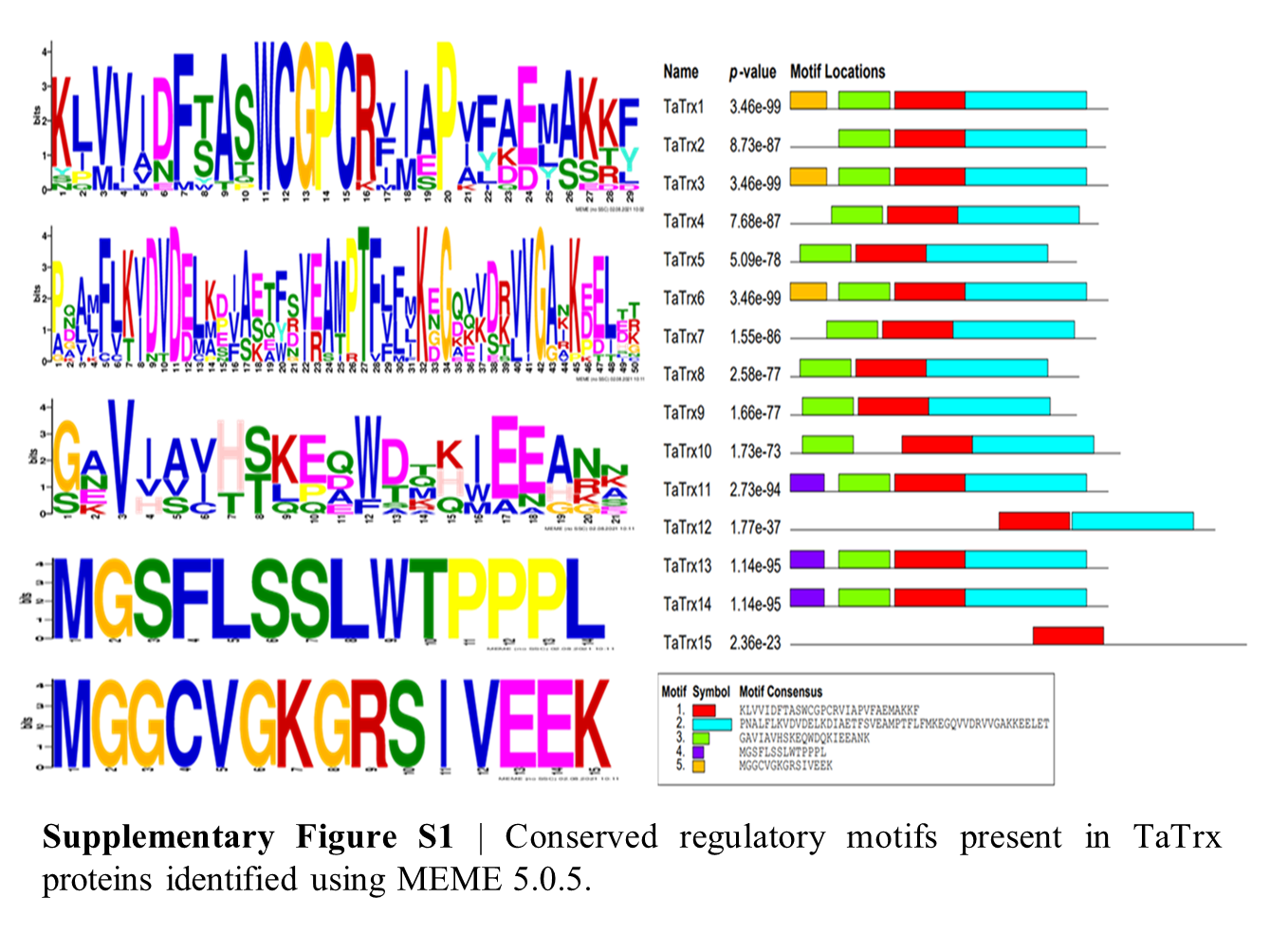

Supplement: Supplementary file 6 [file Image1.TIF]

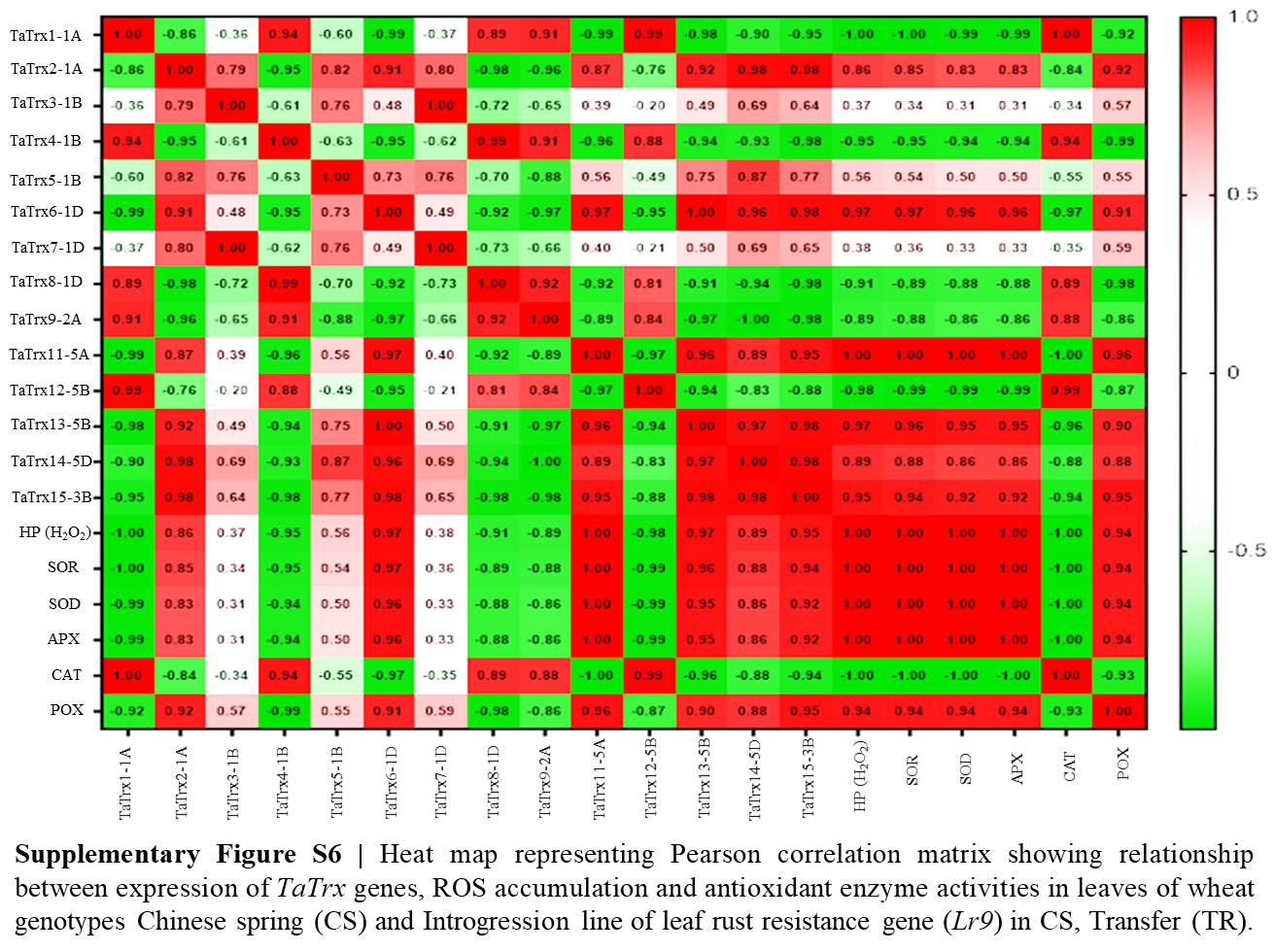

Supplement: Supplementary file 7 [file Image10.TIF]

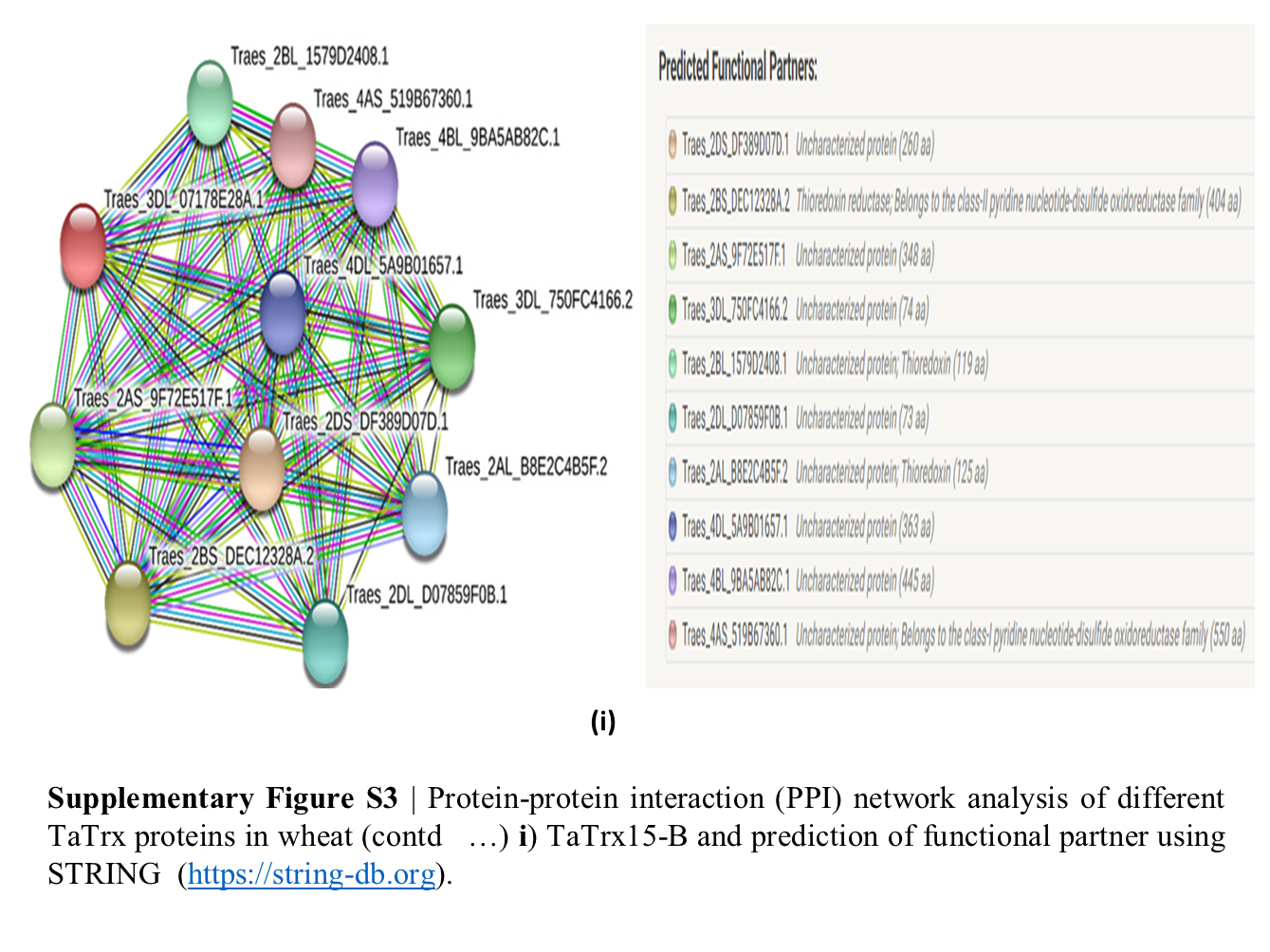

Supplement: Supplementary file 8 [file Image7.TIF]

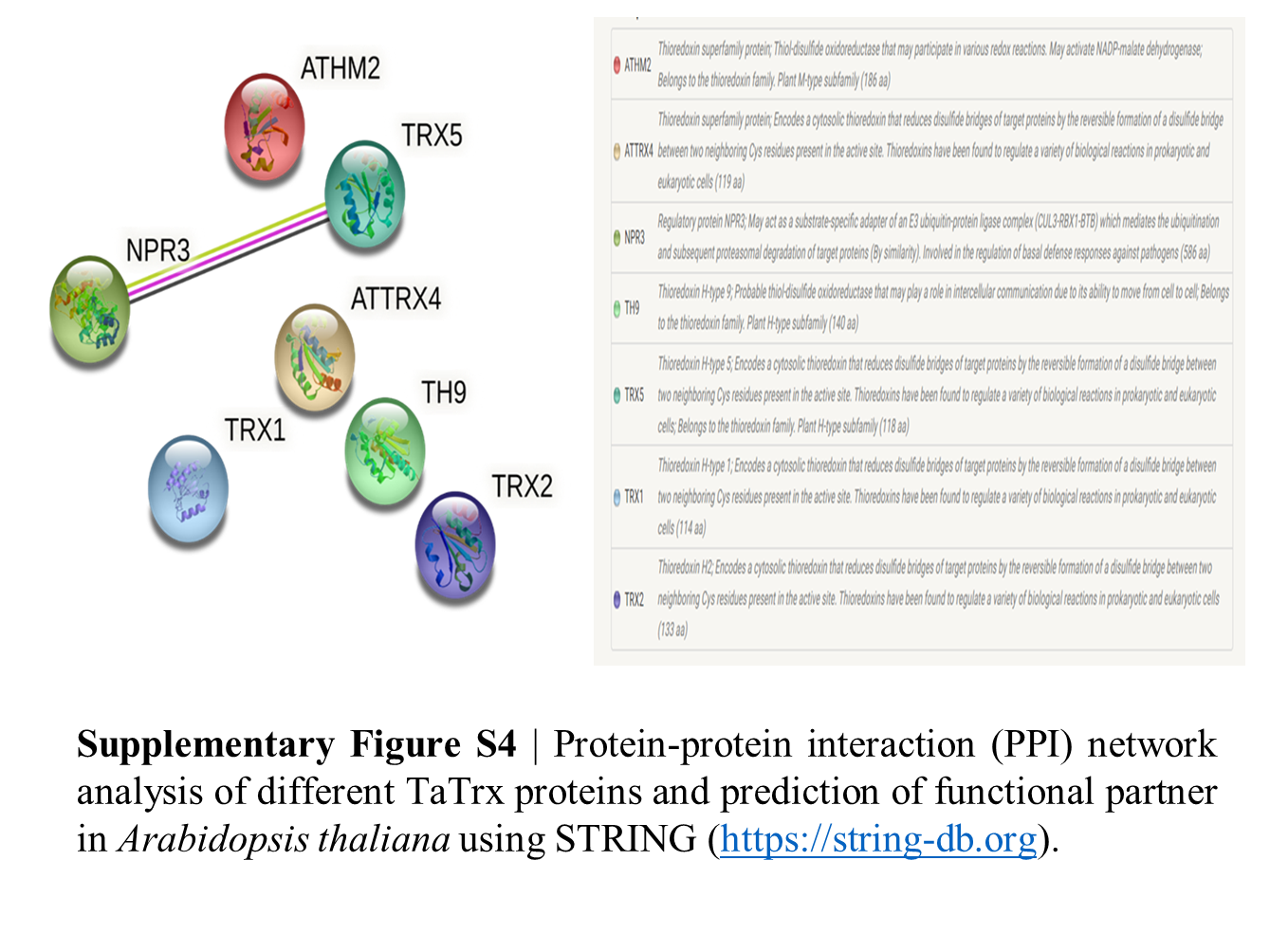

Supplement: Supplementary file 9 [file Image8.TIF]

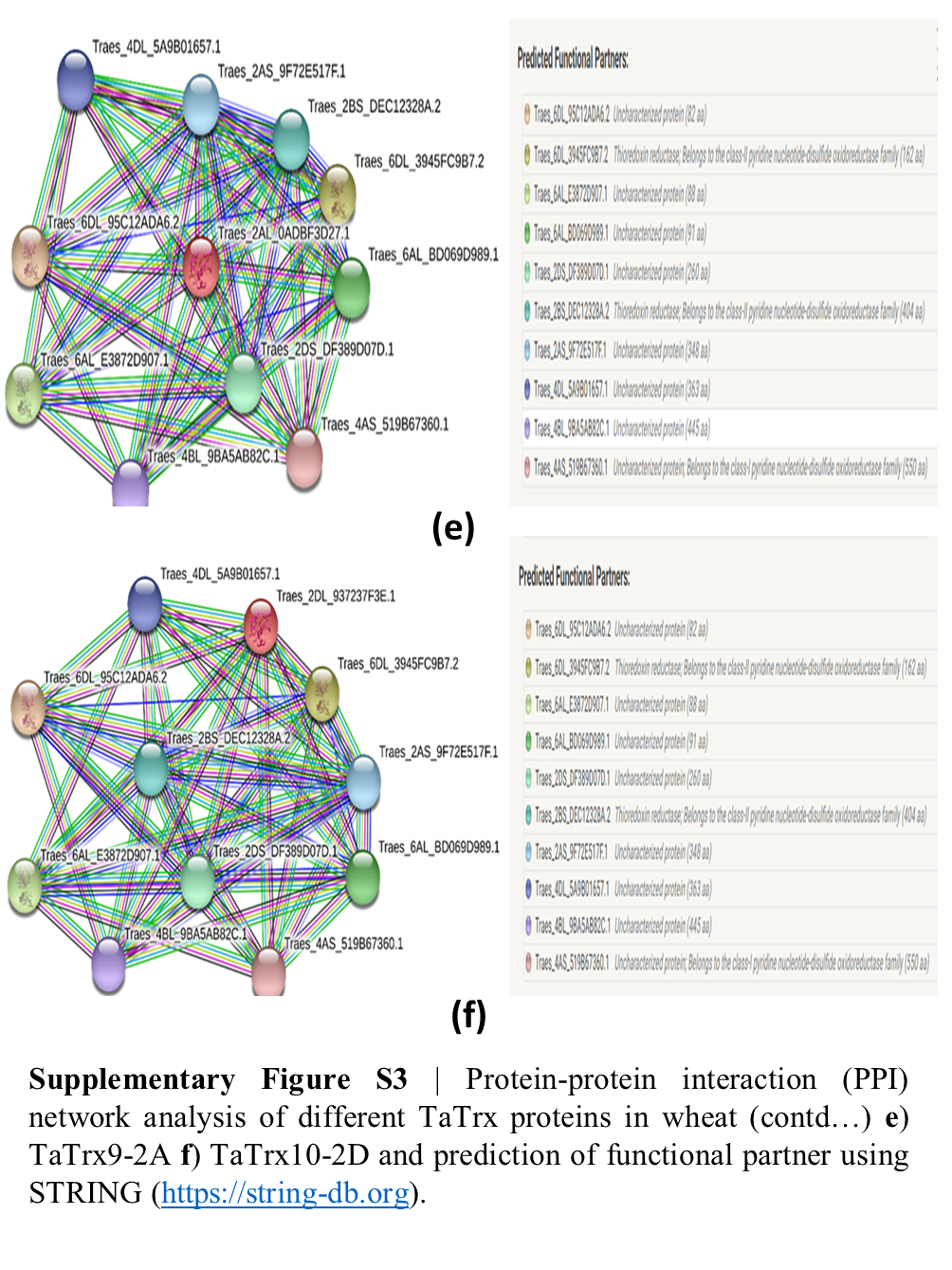

Supplement: Supplementary file 10 [file Image5.TIF]
