# Supplementary material for: Genome-Wide Identification and Expression Analysis of the Thioredoxin (Trx) Gene Family Reveals Its Role in Leaf Rust Resistance in Wheat (Triticum aestivum L.)
Source: Front Genet. 2022 Mar 25;13:836030. doi: 10.3389/fgene.2022.836030 (PMC8990325; doi:10.3389/fgene.2022.836030)
Supplement: Supplementary file 11 [file DataSheet1.docx]

**Table S1** | List of primers used in qRT-PCR for expression analysis.

| **S. no.** | **Primer name** | **Primer sequence** |
| --- | --- | --- |
| 1 | TaTrx1-1A F | TTAATTGCCCGCCAGGAGA |
| 2 | TaTrx1-1A R | CTGACACTGGCAGGAATCG |
| 3 | TaTrx2-1A F | CGCTTGAGAAGTAGTGCCT |
| 4 | TaTrx2-1A R | TACCGGCTTGAGACCACAT |
| 5 | TaTrx3-1B F | ATCCCAGGCCTTCAGGAAC |
| 6 | TaTrx3-1B R | TGGTCCCAGTCCTCTTTGG |
| 7 | TaTrx4-1B F | GATCGAATTCAGTTGTTCGGC |
| 8 | TaTrx4-1B R | GTATAGGCTTGAGATGACATGGA |
| 9 | TaTrx5-1B F | AGCACCACCCAAACTAGCTC |
| 10 | TaTrx5-1B R | CTCCTCGATCTTGGTGCTC |
| 11 | TaTrx6-1D F | GCAAACAGATGCAGCGGTAG |
| 12 | TaTrx6-1D R | TGGTCCCAGTCCTCTTTGG |
| 13 | TaTrx7-1D F | AGCAGTGGACCATGCAGAT |
| 14 | TaTrx7-1D R | TGGCGAGATCAGCGAAAACT |
| 15 | TaTrx8-1D F | TCATGGATAAGCTGGCACTGG |
| 16 | TaTrx8-1D R | TGGACACACGGGGTTTCTTT |
| 17 | TaTrx9-2A F | ACGTTGACGAGCTGAAGGA |
| 18 | TaTrx9-2A R | TGACACAAGAGGGGCATCAC |
| 19 | TaTrx10-2D F | CTGGATTACCAGGTGAGGG |
| 20 | TaTrx10-2D R | TCGTCAACGTCCACCTTCAG |
| 21 | TaTrx11-5A F | TGCTTCGGTTCAGAGTTCGT |
| 22 | TaTrx11-5A R | CCATGGTTCCTTGGCTCTGT |
| 23 | TaTrx12-5B F | ATCCACCGACCAGTTGTGAC |
| 24 | TaTrx12-5B R | CTTTGCTACAGAAAGGATGTCAA |
| 25 | TaTrx13-5B F | CATGAATCAGCCTTGCAGCG |
| 26 | TaTrx13-5B R | GCAAGCCACTAGAAGCAGG |
| 27 | TaTrx14-5D F | TCCTTCCTGTCGTCGTTGTG |
| 28 | TaTrx14-5D R | GTTCCTCCCAGTGCTTGTC |
| 29 | TaTrx15-3B F | GTCTGAGAAGGACCACGACG |
| 30 | TaTrx15-3B R | AATCGCACGCGCTAATTCA |

**Table S2** | List of identified Trx genes present in bread wheat along with their genomic locations.

| **S.no.** | **Gene** | **Ensemble ID** | **Splice Variant** | **Coordinates** | **Length** | | **Exon** | **Coding exon** | **Genome location** |
| --- | --- | --- | --- | --- | --- | --- | --- | --- | --- |
|  |  |  |  |  | **bp** | **aa** |  |  |  |
| 1 | *TaTrx-1* | TraesCS1A02G112400 | 1 | 114,060,494-114,063,211 | 858 | 131 | 5 | 4 | 1A: 114060494 |
| 2 | *TaTrx-2* | TraesCS1A02G325600 | 1 | 516,430,393-516,432,298 | 845 | 130 | 3 | 3 | 1A: 516430393 |
| 3 | *TaTrx-3* | TraesCS1B02G132600 | 1 | 167,108,459-167,111,433 | 1023 | 131 | 5 | 4 | 1B: 167108459 |
| 4 | *TaTrx-4* | TraesCS1B02G338800 | 1 | 566,685,128-566,687,824 | 788 | 127 | 3 | 3 | 1B: 566685128 |
| 5 | *TaTrx-5* | TraesCS1B02G339000 | 1 | 566,823,400-566,831,663 | 742 | 188 | 3 | 3 | 1B: 566823400 |
| 6 | *TaTrx-6* | TraesCS1D02G114000 | 1 | 110,052,054-110,054,117 | 664 | 131 | 5 | 4 | 1D: 110052054 |
| 7 | *TaTrx-7* | TraesCS1D02G327200 | 1 | 419,471,916-419,473,737 | 757 | 126 | 3 | 3 | 1D: 419471916 |
| 8 | *TaTrx-8* | TraesCS1D02G327500 | 1 | 419,717,234-419,723,448 | 637 | 119 | 3 | 3 | 1D: 419717234 |
| 9 | *TaTrx-9* | TraesCS2A02G243200 | 1 | 341,625,205-341,628,292 | 734 | 118 | 4 | 3 | 2A: 341625205 |
| 10 | *TaTrx-10* | TraesCS2D02G244400 | 1 | 274,405,306-274,408,352 | 411 | 136 | 6 | 6 | 2D: 274405306 |
| 11 | *TaTrx-11* | TraesCS5A02G448200 | 1 | 629,709,454-629,713,182 | 899 | 131 | 3 | 3 | 5A: 629709454 |
| 12 | *TaTrx-12* | TraesCS5B02G111800 | 1 | 169,679,181-169,680,177 | 909 | 175 | 2 | 2 | 5B: 169679181 |
| 13 | *TaTrx-13* | TraesCS5B02G452700 | 1 | 625,521,633-625,524,237 | 1268 | 131 | 3 | 3 | 5B: 625521633 |
| 14 | *TaTrx-14* | TraesCS5D02G454800 | 1 | 501,772,372-501,774,724 | 1006 | 131 | 3 | 3 | 5D: 501772372 |
| 15 | *TaTrx-15* | TraesCS3B02G453600 | 1 | 694,324,532-694,325,779 | 971 | 189 | 3 | 3 | 3B: 694324532 |
| 16 | *TaTrx-16* | TraesCS2D02G479600 | 1 | 580,240,075-580,241,653 | 834 | 139 | 3 | 3 | 2D:580240075 |
| 17 | *TaTrx-17* | TraesCS2A02G480300 | 1 | 718,129,055-718,130,574 | 854 | 139 | 3 | 3 | 2A:718129055 |
| 18 | *TaTrx-18* | TraesCS2B02G505100 | 1 | 699,177,857-699,179,469 | 865 | 139 | 3 | 3 | 2B:699177857 |
| 19 | *TaTrx-19* | TraesCS3B02G453600 | 1 | 694,324,532-694,325,779 | 971 | 189 | 3 | 3 | 3B:694324532 |
| 20 | *TaTrx-20* | TraesCS2B02G588600 | 1 | 775,334,949-775,336,794 | 932 | 175 | 2 | 2 | 2B:775334949 |
| 21 | *TaTrx-21* | TraesCS2A02G555300 | 1 | 759,822,758-759,824,170 | 519 | 172 | 2 | 2 | 2A: 759822758 |
| 22 | *TaTrx-22* | TraesCS2D02G559200 | 1 | 631,946,306-631,948,036 | 862 | 173 | 2 | 2 | 2D: 631946306 |
| 23 | *TaTrx-23* | TraesCS2A02G303200 | 1 | 520,301,218-520,307,068 | 1524 | 214 | 5 | 2 | 2A: 520301218 |
| 24 | *TaTrx-24* | TraesCS2D02G301800 | 1 | 385,164,077-385,165,634 | 735 | 244 | 2 | 2 | 2D: 385164077 |
| 25 | *TaTrx-25* | TraesCS2B02G260500 | 1 | 329,138,157-329,138,524 | 288 | 95 | 2 | 2 | 2B: 329138157 |
| 26 | *TaTrx-26* | TraesCS6A02G372100 | 1 | 596,129,766-596,130,697 | 780 | 259 | 3 | 3 | 6A:596129580 |
| 27 | *TaTrx-27* | TraesCS2B02G154700 | 1 | 122,741,144-122,747,015 | 1897 | 520 | 10 | 10 | 2B:122741144 |
| 28 | *TaTrx-28* | TraesCS6B02G409800 | 1 | 684,220,072-684,221,496 | 1425 | 302 | 1 | 1 | 6B:684220072 |
| 29 | *TaTrx-29* | TraesCS6D02G356100 | 2 | 450,614,435-450,615,842 | 1212 | 291 | 2 | 2 | 6D:450614435 |
| 30 | *TaTrx-30* | TraesCS4A02G240000 | 1 | 549,799,338-549,800,138 | 597 | 198 | 3 | 3 | 4A:549799338 |
| 31 | *TaTrx-31* | TraesCS4B02G074900 | 1 | 70,575,735-70,576,619 | 885 | 184 | 1 | 1 | 4B:70575735 |
| 32 | *TaTrx-32* | TraesCS6B02G353700 | 1 | 619,492,681-619,494,113 | 842 | 185 | 5 | 5 | 6B:619492681 |
| 33 | *TaTrx-33* | TraesCS6D02G302900 | 1 | 411,767,774-411,769,316 | 924 | 187 | 5 | 5 | 6D:411767774 |
| 34 | *TaTrx-34* | TraesCS6A02G323100 | 2 | 556,677,509-556,678,983 | 910 | 184 | 5 | 5 | 6A:556677509 |
| 35 | *TaTrx-35* | TraesCS4D02G073500 | 1 | 47,990,881-47,991,812 | 932 | 185 | 1 | 1 | 4D:47990881 |
| 36 | *TaTrx-36* | TraesCS6B02G434200 | 1 | 702,815,241-702,817,606 | 973 | 196 | 5 | 5 | 6B:702815241 |
| 37 | *TaTrx-37* | TraesCS5B02G408100 | 1 | 583,709,780-583,712,961 | 1250 | 267 | 5 | 4 | 5B:583709780 |
| 38 | *TaTrx-38* | TraesCS5A02G403300 | 2 | 595,551,223-595,554,319 | 1183 | 267 | 5 | 4 | 5B:583709780 |
| 39 | *TaTrx-39* | TraesCS6D02G379700 | 2 | 461,333,114-461,335,460 | 964 | 194 | 5 | 5 | 5B:583709780 |
| 40 | *TaTrx-40* | TraesCS5D02G413300 | 1 | 476,194,570-476,197,563 | 1217 | 266 | 5 | 4 | 5B:583709780 |
| 41 | *TaTrx-41* | TraesCS6A02G394200 | 1 | 607,931,222- 607,933,664 | 1044 | 196 | 5 | 5 | 5B:583709780 |
| 42 | *TaTrx-42* | TraesCS6D02G005000 | 1 | 2,269,919-2,272,267 | 1025 | 156 | 6 | 6 | 5B:583709780 |

**Table S3 |** List of identified Trx genes present in different Wheat species.

| ***Triticum urartu*** | | | | | |
| --- | --- | --- | --- | --- | --- |
| **Ensemble/Gene ID** | **Description** | **Genomic Location** | **Orientation** | **Length** | |
|  |  |  |  | **bp** | **aa** |
| TRIUR3_27188 | Thioredoxin-like 1-1, chloroplastic | scaffold68713:47934-48225 | Reverse | 792 | 263 |
| TRIUR3_00652 | Thioredoxin M-type, chloroplastic | scaffold98500:51168-51251 | Forward | 435 | 144 |
| TRIUR3_21265 | Thioredoxin H2-2 | scaffold13060:29524-32048 | Forward | 450 | 149 |
| TRIUR3_18395 | Thioredoxin H-type | scaffold122882:38340-39700 | Reverse | 660 | 219 |
| TRIUR3_12246 | Thioredoxin superfamily protein | scaffold11934:59058-59186 | Forward | 543 | 181 |
| TRIUR3_12708 | Thioredoxin F, chloroplastic | scaffold16114:35201-35326 | Forward | 1353 | 450 |
| TRIUR3_30003 | TPR repeat-containing thioredoxin TTL1 | scaffold25414:46256-46772 | Forward | 1584 | 527 |
| TRIUR3_11378 | Thioredoxin H-type | scaffold74286:47278-47560 | Forward | 384 | 127 |
| TRIUR3_25066 | Thioredoxin domain-containing protein 9-like protein | scaffold276153:1275-3914 | Forward | 825 | 274 |
| TRIUR3_29500 | Thioredoxin-like 1-2, chloroplastic | scaffold21144:30898-31204 | Forward | 771 | 256 |
| TRIUR3_30198 | Thioredoxin | scaffold83505:16206-16510 | Reverse | 411 | 136 |
| TRIUR3_12014 | Thioredoxin-like protein CXXS1 | scaffold13833:44312-45254 | Forward | 417 | 139 |
| TRIUR3_15549 | TPR repeat-containing thioredoxin TDX | scaffold16483:77021-77698 | Forward | 1047 | 348 |
| TRIUR3_11458 | Thioredoxin | scaffold12636:35054-35167 | Forward | 414 | 137 |
| TRIUR3_19546 | TPR repeat-containing thioredoxin TTL1 | scaffold15183:62733-63999 | Reverse | 1494 | 497 |
| TRIUR3_19605 | Thioredoxin | scaffold29763:107642-107773 | Forward | 384 | 128 |
| TRIUR3_23193 | Thioredoxin | scaffold119753:24162-24733 | Reverse | 330 | 110 |
| TRIUR3_30421 | Thioredoxin | scaffold16337:94198-94281 | Reverse | 333 | 110 |
| TRIUR3_17823 | TPR repeat-containing thioredoxin TTL1 | scaffold52734:83049-83575 | Forward | 1620 | 539 |
| TRIUR3_12240 | Thioredoxin H5 | scaffold23248:26930-27795 | Forward | 321 | 106 |
| ***Triticum turgidum*** | | | | | |
| TRITD5Bv1G219670 | Thioredoxin family protein | 5B:621534071-621534238 | Reverse | 237 | 78 |
| TRITD4Av1G066840 | Thioredoxin family protein | 4A:169375621-169375927 | Reverse | 831 | 267 |
| TRITD7Av1G279260 | Thioredoxin | 7A:723500849-723501696 | Forward | 321 | 106 |
| TRITD5Bv1G124350 | Thioredoxin | 5B:368555058-368555848 | Reverse | 378 | 125 |
| TRITD3Bv1G231230 | Thioredoxin | 3B:702423252-702423673 | Reverse | 459 | 152 |
| TRITD1Bv1G222750 | TPR repeat-containing thioredoxin TTL1 | 1B:665169136-665169655 | Reverse | 1221 | 406 |
| TRITD1Av1G225100 | TPR repeat-containing thioredoxin TTL1 | 1A:574021619-574022145 | Reverse | 1224 | 407 |
| TRITD4Av1G066840 | Thioredoxin family protein | 4A:169375621-169375927 | Reverse | 771 | 256 |
| TRITD1Bv1G057630 | Thioredoxin-like protein | 1B:158340129-158341529 | Reverse | 396 | 131 |
| TRITD2Av1G123680 | Thioredoxin | 2A:337140197-337140501 | Forward | 288 | 95 |
| TRITD3Av1G000740 | Thioredoxin | 3A:1412855-1413137 | Reverse | 384 | 127 |
| TRITD3Bv1G003730 | Thioredoxin | 3B:6970958-6971298 | Reverse | 372 | 123 |
| TRITD3Bv1G231230 | Thioredoxin | 3B:702423361-702423673 | Reverse | 570 | 189 |
| TRITD5Bv1G219670 | Thioredoxin family protein | 5B:621532824-621534413 | Reverse | 396 | 131 |
| TRITD1Av1G225100 | TPR repeat-containing thioredoxin TTL1 | 1A:574021619-574022145 | Reverse | 1971 | 656 |
| TRITD1Bv1G065790 | Thioredoxin | 1B:183156146-183162380 | Forward | 648 | 215 |
| TRITD1Av1G191890 | Thioredoxin | 1A:507933744-507935125 | Forward | 393 | 130 |
| TRITD5Bv1G124350 | Thioredoxin | 5B:368555058-368555848 | Reverse | 960 | 319 |
| TRITD1Bv1G222750 | TPR repeat-containing thioredoxin TTL1 | 1B:665169136-665169655 | Reverse | 1962 | 653 |
| TRITD2Bv1G034490 | Thioredoxin, putative | 2B:83399042-83399334 | Reverse | 822 | 273 |
| TRITD1Av1G059400 | Thioredoxin | 1A:144064087-144067516 | Reverse | 648 | 215 |
| TRITD1Bv1G181650 | Thioredoxin | 1B:559627705-559629932 | Forward | 384 | 127 |
| TRITD2Av1G264730 | Thioredoxin | 2A:712450261-712451193 | Reverse | 420 | 139 |
| TRITD3Bv1G231230 | Thioredoxin | 3B:702423361-702423673 | Reverse | 357 | 118 |
| TRITD5Bv1G219670 | Thioredoxin family protein | 5B:621532824-621534238 | Reverse | 441 | 146 |
| TRITD4Av1G066840 | Thioredoxin family protein | 4A:169375621-169375927 | Reverse | 582 | 193 |
| TRITD2Bv1G112850 | Thioredoxin | 2B:329100011-329100315 | Reverse | 288 | 95 |
| TRITD3Av1G000740 | Thioredoxin | 3A:1412855-1413137 | Reverse | 369 | 122 |
| TRITD2Av1G123680 | Thioredoxin | 2A:337140197-337140501 | Forward | 411 | 136 |
| TRITD1Bv1G057630 | Thioredoxin-like protein | 1B:158340129-158341529 | Reverse | 438 | 145 |
| TRITD2Bv1G227440 | Thioredoxin | 2B:686252713-686253721 | Reverse | 420 | 139 |
| TRITD3Bv1G003690 | Thioredoxin | 3B:6886409-6886734 | Forward | 372 | 123 |
| ***Aegilops tauschii*** | | | | | |
| AET2Gv20534200 | H-type thioredoxin, , Stress response | 2D:272782420-272782539 | Forward | 878 | 51 |
| AET1Gv20787800 |  | 1D:426404863-426412833 | Forward | 833 | 125 |
| AET5Gv21035800 |  | 5D:513854572-513857917 | Reverse | 1075 | 106 |
| AET1Gv20280000 |  | 1D:112529793-112530017 | Reverse | 863 | 59 |
| AET5Gv20500700 |  | 5D:326716102-326716437 | Reverse | 734 | 125 |
| AET2Gv21058400 |  | 2D:578495925-578496956 | Reverse | 999 | 139 |
| AET3Gv20017500 |  | 3D:3041492-3041746 | Forward | 3779 | 1074 |
| AET1Gv20478300 |  | 1D:264500092-264500181 | Forward | 3901 | 136 |
| AET6Gv20916500 | Thioredoxin domain 2 containing protein | 6D:473096627-473097076 | Forward | 1658 | 303 |
| AET4Gv20154400 | Thioredoxin-like protein CITRX, chloroplastic | 4D:50799120-50799488 | Forward | 996 | 223 |

**Table S4** | Details of identified conserved regulatory motifs present in Trx protein.

| **Discovered motif** | **E-value** | **Sites** | **Width** | **Log Likelihood Ratio** | **Information Content** | **Relative Entropy** | **Bayes Threshold** |
| --- | --- | --- | --- | --- | --- | --- | --- |
| KLVVIDFTASWCGPCRVIAPVFAEMAKKF | 1.10E-23 | 15 | 29 | 980 | 98.1 | 94.3 | 6.7261 |
| PNALFLKVDVDELKDIAETFSVEAMPTFLFMKEGQVVDRVVGAKKEELET | 5.40E-274 | 14 | 50 | 1370 | 157.4 | 142.2 | 8.1884 |
| GAVIAVHSKEQWDQKIEEANK | 2.60E-94 | 13 | 21 | 555 | 62.6 | 61.6 | 8.6768 |
| MGSFLSSLWTPPPL | 1.00E-12 | 3 | 14 | 127 | 60.5 | 60.9 | 9.69681 |
| MGGCVGKGRSIVEEK | 1.10E-08 | 3 | 15 | 127 | 64.8 | 61 | 9.68491 |

**Table S5** | Putative cis-elements of Wheat *TaTrx* genes identified using PlantCARE database.

| **Gene** | **Promoter** | **Consensus sequence** | **Position** | | **Strand** | **Function** |
| --- | --- | --- | --- | --- | --- | --- |
|  |  |  | **Start** | **End** |  |  |
| TaTrx1-1A | ABRE | GCCGCGTGGC | 97 | 106 | - | cis-acting element involved in the abscisic acid responsiveness |
| TaTrx1-1A | ABRE | GCAACGTGTC | 538 | 547 | + | cis-acting element involved in the abscisic acid responsiveness |
| TaTrx1-1A | TGACG-motif | TGACG | 468 | 473 | + | cis-acting regulatory element involved in the MeJA-responsiveness |
| TaTrx1-1A | TC-rich repeats | GTTTTCTTAC | 898 | 907 | - | cis-acting element involved in defense and stress responsiveness |
| TaTrx1-1A | CGTCA-motif | CGTCA | 468 | 473 | - | cis-acting regulatory element involved in the MeJA-responsiveness |
| TaTrx1-1A | TCA-element | CCATCTTTTT | 547 | 556 | + | cis-acting element involved in salicylic acid responsiveness |
| TaTrx2-1A | TGACG-motif | TGACG | 1433 | 1438 | - | cis-acting regulatory element involved in the MeJA-responsiveness |
| TaTrx2-1A | TGACG-motif | TGACG | 1471 | 1476 | + | cis-acting regulatory element involved in the MeJA-responsiveness |
| TaTrx2-1A | ABRE | ACGTG | 778 | 783 | + | cis-acting element involved in the abscisic acid responsiveness |
| TaTrx2-1A | ABRE | ACGTG | 964 | 969 | - | cis-acting element involved in the abscisic acid responsiveness |
| TaTrx2-1A | ABRE | GCCGCGTGGC | 1489 | 1498 | - | cis-acting element involved in the abscisic acid responsiveness |
| TaTrx2-1A | CGTCA-motif | CGTCA | 605 | 610 | + | cis-acting regulatory element involved in the MeJA-responsiveness |
| TaTrx2-1A | CGTCA-motif | CGTCA | 1433 | 1438 | + | cis-acting regulatory element involved in the MeJA-responsiveness |
| TaTrx2-1A | CGTCA-motif | CGTCA | 1471 | 1476 | - | cis-acting regulatory element involved in the MeJA-responsiveness |
| TaTrx3-1B | TC-rich repeats | GTTTTCTTAC | 1306 | 1315 | - | cis-acting element involved in defense and stress responsiveness |
| TaTrx3-1B | ABRE | ACGTG | 6 | 11 | + | cis-acting element involved in the abscisic acid responsiveness |
| TaTrx3-1B | ABRE | ACGTG | 72 | 77 | + | cis-acting element involved in the abscisic acid responsiveness |
| TaTrx3-1B | ABRE | CACGTG | 99 | 105 | + | cis-acting element involved in the abscisic acid responsiveness |
| TaTrx3-1B | ABRE | ACGTG | 100 | 105 | + | cis-acting element involved in the abscisic acid responsiveness |
| TaTrx3-1B | ABRE | GCCGCGTGGC | 230 | 239 | - | cis-acting element involved in the abscisic acid responsiveness |
| TaTrx3-1B | ABRE | GCAACGTGTC | 690 | 699 | + | cis-acting element involved in the abscisic acid responsiveness |
| TaTrx3-1B | ABRE | ACGTG | 2341 | 2346 | + | cis-acting element involved in the abscisic acid responsiveness |
| TaTrx4-1B | CGTCA-motif | CGTCA | 595 | 600 | + | cis-acting regulatory element involved in the MeJA-responsiveness |
| TaTrx4-1B | CGTCA-motif | CGTCA | 1053 | 1058 | + | cis-acting regulatory element involved in the MeJA-responsiveness |
| TaTrx4-1B | CGTCA-motif | CGTCA | 1113 | 1118 | + | cis-acting regulatory element involved in the MeJA-responsiveness |
| TaTrx4-1B | CGTCA-motif | CGTCA | 1971 | 1976 | + | cis-acting regulatory element involved in the MeJA-responsiveness |
| TaTrx4-1B | CGTCA-motif | CGTCA | 2314 | 2319 | - | cis-acting regulatory element involved in the MeJA-responsiveness |
| TaTrx4-1B | TGACG-motif | TGACG | 595 | 600 | - | cis-acting regulatory element involved in the MeJA-responsiveness |
| TaTrx4-1B | TGACG-motif | TGACG | 1053 | 1058 | - | cis-acting regulatory element involved in the MeJA-responsiveness |
| TaTrx4-1B | TGACG-motif | TGACG | 1113 | 1118 | - | cis-acting regulatory element involved in the MeJA-responsiveness |
| TaTrx4-1B | TGACG-motif | TGACG | 1971 | 1976 | - | cis-acting regulatory element involved in the MeJA-responsiveness |
| TaTrx4-1B | TGACG-motif | TGACG | 2314 | 2319 | + | cis-acting regulatory element involved in the MeJA-responsiveness |
| TaTrx4-1B | ABRE | ACGTG | 1051 | 1056 | - | cis-acting element involved in the abscisic acid responsiveness |
| TaTrx4-1B | ABRE | ACGTG | 1278 | 1283 | - | cis-acting element involved in the abscisic acid responsiveness |
| TaTrx5-1B | CGTCA-motif | CGTCA | 1819 | 1824 | + | cis-acting regulatory element involved in the MeJA-responsiveness |
| TaTrx5-1B | CGTCA-motif | CGTCA | 3199 | 3204 | + | cis-acting regulatory element involved in the MeJA-responsiveness |
| TaTrx5-1B | CGTCA-motif | CGTCA | 3978 | 3983 | + | cis-acting regulatory element involved in the MeJA-responsiveness |
| TaTrx5-1B | CGTCA-motif | CGTCA | 4982 | 4987 | - | cis-acting regulatory element involved in the MeJA-responsiveness |
| TaTrx5-1B | CGTCA-motif | CGTCA | 6085 | 6090 | + | cis-acting regulatory element involved in the MeJA-responsiveness |
| TaTrx5-1B | CGTCA-motif | CGTCA | 7160 | 7165 | + | cis-acting regulatory element involved in the MeJA-responsiveness |
| TaTrx5-1B | ABRE | AACCCGG | 570 | 577 | + | cis-acting element involved in the abscisic acid responsiveness |
| TaTrx5-1B | ABRE | ACGTG | 623 | 628 | - | cis-acting element involved in the abscisic acid responsiveness |
| TaTrx5-1B | ABRE | ACGTG | 1415 | 1420 | + | cis-acting element involved in the abscisic acid responsiveness |
| TaTrx5-1B | ABRE | TACGGTC | 1861 | 1868 | + | cis-acting element involved in the abscisic acid responsiveness |
| TaTrx5-1B | ABRE | TACGGTC | 4541 | 4548 | + | cis-acting element involved in the abscisic acid responsiveness |
| TaTrx5-1B | ABRE | CACGTG | 4860 | 4866 | - | cis-acting element involved in the abscisic acid responsiveness |
| TaTrx5-1B | ABRE | ACGTG | 4861 | 4866 | + | cis-acting element involved in the abscisic acid responsiveness |
| TaTrx5-1B | ABRE | ACGTG | 5100 | 5105 | + | cis-acting element involved in the abscisic acid responsiveness |
| TaTrx5-1B | ABRE | CACGTG | 6218 | 6224 | - | cis-acting element involved in the abscisic acid responsiveness |
| TaTrx5-1B | ABRE | ACGTG | 6219 | 6224 | + | cis-acting element involved in the abscisic acid responsiveness |
| TaTrx5-1B | ABRE | ACGTG | 8095 | 8100 | - | cis-acting element involved in the abscisic acid responsiveness |
| TaTrx5-1B | TCA-element | TCAGAAGAGG | 37 | 46 | + | cis-acting element involved in salicylic acid responsiveness |
| TaTrx5-1B | TCA-element | CCATCTTTTT | 1089 | 1098 | - | cis-acting element involved in salicylic acid responsiveness |
| TaTrx5-1B | TC-rich repeats | ATTCTCTAAC | 5982 | 5991 | - | cis-acting element involved in defense and stress responsiveness |
| TaTrx5-1B | TC-rich repeats | GTTTTCTTAC | 6579 | 6588 | + | cis-acting element involved in defense and stress responsiveness |
| TaTrx5-1B | TGACG-motif | TGACG | 734 | 739 | + | cis-acting regulatory element involved in the MeJA-responsiveness |
| TaTrx5-1B | TGACG-motif | TGACG | 1819 | 1824 | - | cis-acting regulatory element involved in the MeJA-responsiveness |
| TaTrx5-1B | TGACG-motif | TGACG | 3199 | 3204 | - | cis-acting regulatory element involved in the MeJA-responsiveness |
| TaTrx5-1B | TGACG-motif | TGACG | 3978 | 3983 | - | cis-acting regulatory element involved in the MeJA-responsiveness |
| TaTrx5-1B | TGACG-motif | TGACG | 4982 | 4987 | + | cis-acting regulatory element involved in the MeJA-responsiveness |
| TaTrx5-1B | TGACG-motif | TGACG | 6085 | 6090 | - | cis-acting regulatory element involved in the MeJA-responsiveness |
| TaTrx5-1B | TGACG-motif | TGACG | 7160 | 7165 | - | cis-acting regulatory element involved in the MeJA-responsiveness |
| TaTrx6-1D | ABRE | GCAACGTGTC | 152 | 161 | + | cis-acting element involved in the abscisic acid responsiveness |
| TaTrx6-1D | CGTCA-motif | CGTCA | 82 | 87 | - | cis-acting regulatory element involved in the MeJA-responsiveness |
| TaTrx6-1D | TC-rich repeats | GTTTTCTTAC | 490 | 499 | - | cis-acting element involved in defense and stress responsiveness |
| TaTrx6-1D | TCA-element | CCATCTTTTT | 161 | 170 | + | cis-acting element involved in salicylic acid responsiveness |
| TaTrx7-1D | TGACG-motif | TGACG | 1459 | 1464 | + | cis-acting regulatory element involved in the MeJA-responsiveness |
| TaTrx7-1D | ABRE | TACGGTC | 359 | 366 | + | cis-acting element involved in the abscisic acid responsiveness |
| TaTrx7-1D | ABRE | CACGTG | 1012 | 1018 | - | cis-acting element involved in the abscisic acid responsiveness |
| TaTrx7-1D | ABRE | ACGTG | 1013 | 1018 | + | cis-acting element involved in the abscisic acid responsiveness |
| TaTrx7-1D | CGTCA-motif | CGTCA | 1459 | 1464 | - | cis-acting regulatory element involved in the MeJA-responsiveness |
| TaTrx8-1D | ABRE | AACCCGG | 474 | 481 | + | cis-acting element involved in the abscisic acid responsiveness |
| TaTrx8-1D | ABRE | ACGTG | 527 | 532 | - | cis-acting element involved in the abscisic acid responsiveness |
| TaTrx8-1D | ABRE | CGTACGTGCA | 623 | 632 | - | cis-acting element involved in the abscisic acid responsiveness |
| TaTrx8-1D | ABRE | ACGTG | 662 | 667 | - | cis-acting element involved in the abscisic acid responsiveness |
| TaTrx8-1D | ABRE | CACGTG | 2244 | 2250 | + | cis-acting element involved in the abscisic acid responsiveness |
| TaTrx8-1D | ABRE | ACGTG | 2245 | 2250 | + | cis-acting element involved in the abscisic acid responsiveness |
| TaTrx8-1D | ABRE | CGTACGTGCA | 2455 | 2465 | + | cis-acting element involved in the abscisic acid responsiveness |
| TaTrx8-1D | ABRE | ACGTG | 2458 | 2463 | + | cis-acting element involved in the abscisic acid responsiveness |
| TaTrx8-1D | ABRE | ACGTG | 2580 | 2585 | - | cis-acting element involved in the abscisic acid responsiveness |
| TaTrx8-1D | ABRE | ACGTG | 2627 | 2632 | - | cis-acting element involved in the abscisic acid responsiveness |
| TaTrx8-1D | ABRE | ACGTG | 3429 | 3434 | - | cis-acting element involved in the abscisic acid responsiveness |
| TaTrx8-1D | TCA-element | CCATCTTTTT | 991 | 1000 | - | cis-acting element involved in salicylic acid responsiveness |
| TaTrx8-1D | TCA-element | CCATCTTTTT | 1535 | 1544 | + | cis-acting element involved in salicylic acid responsiveness |
| TaTrx8-1D | TCA-element | CCATCTTTTT | 3792 | 3801 | + | cis-acting element involved in salicylic acid responsiveness |
| TaTrx8-1D | CGTCA-motif | CGTCA | 646 | 651 | - | cis-acting regulatory element involved in the MeJA-responsiveness |
| TaTrx8-1D | CGTCA-motif | CGTCA | 664 | 669 | + | cis-acting regulatory element involved in the MeJA-responsiveness |
| TaTrx8-1D | CGTCA-motif | CGTCA | 1999 | 2004 | + | cis-acting regulatory element involved in the MeJA-responsiveness |
| TaTrx8-1D | CGTCA-motif | CGTCA | 3049 | 3054 | - | cis-acting regulatory element involved in the MeJA-responsiveness |
| TaTrx8-1D | CGTCA-motif | CGTCA | 5133 | 5138 | + | cis-acting regulatory element involved in the MeJA-responsiveness |
| TaTrx8-1D | TC-rich repeats | GTTTTCTTAC | 603 | 612 | + | cis-acting element involved in defense and stress responsiveness |
| TaTrx8-1D | TC-rich repeats | ATTCTCTAAC | 4212 | 4221 | - | cis-acting element involved in defense and stress responsiveness |
| TaTrx8-1D | TC-rich repeats | GTTTTCTTAC | 4552 | 4561 | + | cis-acting element involved in defense and stress responsiveness |
| TaTrx8-1D | TC-rich repeats | ATTCTCTAAC | 5967 | 5976 | - | cis-acting element involved in defense and stress responsiveness |
| TaTrx8-1D | TGACG-motif | TGACG | 646 | 651 | + | cis-acting regulatory element involved in the MeJA-responsiveness |
| TaTrx8-1D | TGACG-motif | TGACG | 664 | 669 | - | cis-acting regulatory element involved in the MeJA-responsiveness |
| TaTrx8-1D | TGACG-motif | TGACG | 1999 | 2004 | - | cis-acting regulatory element involved in the MeJA-responsiveness |
| TaTrx8-1D | TGACG-motif | TGACG | 3049 | 3054 | + | cis-acting regulatory element involved in the MeJA-responsiveness |
| TaTrx8-1D | TGACG-motif | TGACG | 5133 | 5138 | - | cis-acting regulatory element involved in the MeJA-responsiveness |
| TaTrx9-2A | TGACG-motif | TGACG | 1898 | 1903 | + | cis-acting regulatory element involved in the MeJA-responsiveness |
| TaTrx9-2A | TGACG-motif | TGACG | 2389 | 2394 | + | cis-acting regulatory element involved in the MeJA-responsiveness |
| TaTrx9-2A | ABRE | ACGTG | 1176 | 1181 | + | cis-acting element involved in the abscisic acid responsiveness |
| TaTrx9-2A | TCA-element | CCATCTTTTT | 1799 | 1808 | + | cis-acting element involved in salicylic acid responsiveness |
| TaTrx9-2A | TCA-element | CCATCTTTTT | 1854 | 1863 | + | cis-acting element involved in salicylic acid responsiveness |
| TaTrx9-2A | CGTCA-motif | CGTCA | 1898 | 1903 | - | cis-acting regulatory element involved in the MeJA-responsiveness |
| TaTrx9-2A | CGTCA-motif | CGTCA | 2389 | 2394 | - | cis-acting regulatory element involved in the MeJA-responsiveness |
| TaTrx10-2D | TCA-element | CCATCTTTTT | 1700 | 1709 | + | cis-acting element involved in salicylic acid responsiveness |
| TaTrx10-2D | TCA-element | CCATCTTTTT | 1755 | 1764 | + | cis-acting element involved in salicylic acid responsiveness |
| TaTrx10-2D | TGACG-motif | TGACG | 385 | 390 | + | cis-acting regulatory element involved in the MeJA-responsiveness |
| TaTrx10-2D | TGACG-motif | TGACG | 1799 | 1804 | + | cis-acting regulatory element involved in the MeJA-responsiveness |
| TaTrx10-2D | TGACG-motif | TGACG | 2751 | 2756 | + | cis-acting regulatory element involved in the MeJA-responsiveness |
| TaTrx10-2D | CGTCA-motif | CGTCA | 385 | 390 | - | cis-acting regulatory element involved in the MeJA-responsiveness |
| TaTrx10-2D | CGTCA-motif | CGTCA | 1799 | 1804 | - | cis-acting regulatory element involved in the MeJA-responsiveness |
| TaTrx10-2D | CGTCA-motif | CGTCA | 2751 | 2756 | - | cis-acting regulatory element involved in the MeJA-responsiveness |
| TaTrx11-5A | ABRE | GCCGCGTGGC | 1421 | 1430 | - | cis-acting element involved in the abscisic acid responsiveness |
| TaTrx11-5A | ABRE | CGTACGTGCA | 1878 | 1887 | - | cis-acting element involved in the abscisic acid responsiveness |
| TaTrx11-5A | ABRE | ACGTG | 1880 | 1885 | - | cis-acting element involved in the abscisic acid responsiveness |
| TaTrx11-5A | ABRE | GCCGCGTGGC | 1923 | 1932 | + | cis-acting element involved in the abscisic acid responsiveness |
| TaTrx11-5A | ABRE | GCCGCGTGGC | 1943 | 1952 | - | cis-acting element involved in the abscisic acid responsiveness |
| TaTrx11-5A | ABRE | ACGTG | 1969 | 1974 | + | cis-acting element involved in the abscisic acid responsiveness |
| TaTrx11-5A | ABRE | GCCGCGTGGC | 2046 | 2055 | + | cis-acting element involved in the abscisic acid responsiveness |
| TaTrx11-5A | ABRE | GCCGCGTGGC | 2182 | 2191 | + | cis-acting element involved in the abscisic acid responsiveness |
| TaTrx11-5A | ABRE | ACGTG | 2741 | 2746 | - | cis-acting element involved in the abscisic acid responsiveness |
| TaTrx11-5A | CGTCA-motif | CGTCA | 406 | 411 | - | cis-acting regulatory element involved in the MeJA-responsiveness |
| TaTrx11-5A | CGTCA-motif | CGTCA | 539 | 544 | + | cis-acting regulatory element involved in the MeJA-responsiveness |
| TaTrx11-5A | CGTCA-motif | CGTCA | 764 | 769 | - | cis-acting regulatory element involved in the MeJA-responsiveness |
| TaTrx11-5A | TC-rich repeats | GTTTTCTTAC | 3258 | 3267 | + | cis-acting element involved in defense and stress responsiveness |
| TaTrx11-5A | TGACG-motif | TGACG | 406 | 411 | + | cis-acting regulatory element involved in the MeJA-responsiveness |
| TaTrx11-5A | TGACG-motif | TGACG | 539 | 544 | - | cis-acting regulatory element involved in the MeJA-responsiveness |
| TaTrx11-5A | TGACG-motif | TGACG | 764 | 769 | + | cis-acting regulatory element involved in the MeJA-responsiveness |
| TaTrx12-5B | ABRE | ACGTG | 487 | 492 | + | cis-acting element involved in the abscisic acid responsiveness |
| TaTrx12-5B | ABRE | ACGTG | 836 | 841 | - | cis-acting element involved in the abscisic acid responsiveness |
| TaTrx12-5B | TGACG-motif | TGACG | 362 | 367 | + | cis-acting regulatory element involved in the MeJA-responsiveness |
| TaTrx12-5B | TGACG-motif | TGACG | 608 | 613 | - | cis-acting regulatory element involved in the MeJA-responsiveness |
| TaTrx12-5B | TGACG-motif | TGACG | 838 | 843 | - | cis-acting regulatory element involved in the MeJA-responsiveness |
| TaTrx12-5B | CGTCA-motif | CGTCA | 362 | 367 | - | cis-acting regulatory element involved in the MeJA-responsiveness |
| TaTrx12-5B | CGTCA-motif | CGTCA | 608 | 613 | + | cis-acting regulatory element involved in the MeJA-responsiveness |
| TaTrx12-5B | CGTCA-motif | CGTCA | 838 | 843 | + | cis-acting regulatory element involved in the MeJA-responsiveness |
| TaTrx13-5B | TGACG-motif | TGACG | 239 | 244 | + | cis-acting regulatory element involved in the MeJA-responsiveness |
| TaTrx13-5B | TGACG-motif | TGACG | 375 | 380 | - | cis-acting regulatory element involved in the MeJA-responsiveness |
| TaTrx13-5B | TGACG-motif | TGACG | 558 | 563 | + | cis-acting regulatory element involved in the MeJA-responsiveness |
| TaTrx13-5B | TGACG-motif | TGACG | 641 | 646 | - | cis-acting regulatory element involved in the MeJA-responsiveness |
| TaTrx13-5B | ABRE | ACGTG | 1092 | 1097 | - | cis-acting element involved in the abscisic acid responsiveness |
| TaTrx13-5B | CGTCA-motif | CGTCA | 239 | 244 | - | cis-acting regulatory element involved in the MeJA-responsiveness |
| TaTrx13-5B | CGTCA-motif | CGTCA | 375 | 380 | + | cis-acting regulatory element involved in the MeJA-responsiveness |
| TaTrx13-5B | CGTCA-motif | CGTCA | 558 | 563 | - | cis-acting regulatory element involved in the MeJA-responsiveness |
| TaTrx13-5B | CGTCA-motif | CGTCA | 641 | 646 | + | cis-acting regulatory element involved in the MeJA-responsiveness |
| TaTrx13-5B | TC-rich repeats | GTTTTCTTAC | 1609 | 1618 | + | cis-acting element involved in defense and stress responsiveness |
| TaTrx13-5B | TC-rich repeats | GTTTTCTTAC | 2059 | 2068 | + | cis-acting element involved in defense and stress responsiveness |
| TaTrx14-5D | ABRE | ACGTG | 1247 | 1252 | - | cis-acting element involved in the abscisic acid responsiveness |
| TaTrx14-5D | TC-rich repeats | GTTTTCTTAC | 1765 | 1774 | + | cis-acting element involved in defense and stress responsiveness |
| TaTrx14-5D | TC-rich repeats | GTTTTCTTAC | 2215 | 2224 | + | cis-acting element involved in defense and stress responsiveness |
| TaTrx14-5D | CGTCA-motif | CGTCA | 396 | 401 | - | cis-acting regulatory element involved in the MeJA-responsiveness |
| TaTrx14-5D | CGTCA-motif | CGTCA | 529 | 534 | + | cis-acting regulatory element involved in the MeJA-responsiveness |
| TaTrx14-5D | CGTCA-motif | CGTCA | 795 | 800 | + | cis-acting regulatory element involved in the MeJA-responsiveness |
| TaTrx14-5D | TGACG-motif | TGACG | 396 | 401 | + | cis-acting regulatory element involved in the MeJA-responsiveness |
| TaTrx14-5D | TGACG-motif | TGACG | 529 | 534 | - | cis-acting regulatory element involved in the MeJA-responsiveness |
| TaTrx14-5D | TGACG-motif | TGACG | 795 | 800 | - | cis-acting regulatory element involved in the MeJA-responsiveness |
| TaTrx14-5D | TCA-element | CCATCTTTTT | 188 | 197 | - | cis-acting element involved in salicylic acid responsiveness |
| TaTrx15-3B | CGTCA-motif | CGTCA | 431 | 436 | + | cis-acting regulatory element involved in the MeJA-responsiveness |
| TaTrx15-3B | TCA-element | TCAGAAGAGG | 759 | 769 | + | cis-acting element involved in salicylic acid responsiveness |
| TaTrx15-3B | TGACG-motif | TGACG | 431 | 436 | - | cis-acting regulatory element involved in the MeJA-responsiveness |
| TaTrx15-3B | ABRE | ACGTG | 467 | 472 | - | cis-acting element involved in the abscisic acid responsiveness |
| TaTrx15-3B | ABRE | ACGTG | 1987 | 1992 | - | cis-acting element involved in the abscisic acid responsiveness |
| TaTrx15-3B | TC-rich repeats | ATTCTCTAAC | 2061 | 2070 | + | cis-acting element involved in defense and stress responsiveness |

**Table S6** | List of identified putative miRNA targets for *TaTrx* genes.

| **miRNA Acc.** | **Target Gene** |  | **Target start** | **Target end** | **miRNA aligned fragment** | **Target aligned fragment** | **Inhibition** |
| --- | --- | --- | --- | --- | --- | --- | --- |
| tae-miR9673-5p | TaTrx2-1A |  | 583 | 602 | UAAGAAGCAAAUAGCACAUG | UGUGUACUGCUUGCUUCUUG | Translation |
| tae-miR9658-3p | TaTrx9-2A |  | 358 | 378 | AUCGUUCUGGGUGAAUAGGCC | UUCCUGUUUAUCAAGGAUGGU | Cleavage |
| tae-miR9658-3p | TaTrx10-2D |  | 307 | 327 | AUCGUUCUGGGUGAAUAGGCC | UUCCUGUUUAUCAAGGAUGGU | Cleavage |
| tae-miR9674a-5p | TaTrx10-2D |  | 348 | 368 | GCAUCAUCCAUCCUACCAUUC | CGGUGGUAGGAAGGAUGAUAU | Translation |
| tae-miR1136 | TaTrx5-1B |  | 625 | 648 | UUGUCGCAGGUAUGGAUGUAUCUA | GCGGCGCACACGUACUGGCGACAA | Cleavage |
| tae-miR396-5p | TaTrx2-1A |  | 595 | 615 | AACUGUGAACUCGCGGGGAUG | GCUUCUUGUGAGUUUAUGGAU | Cleavage |
| tae-miR9666a-3p | TaTrx13-5B |  | 585 | 606 | CGGUAGGGCUGUAUGAUGGCGA | UCCCCAUGGUGUAGCACUACCA | Cleavage |
| tae-miR9674b-5p | TaTrx7-1D |  | 584 | 604 | AUAGCAUCAUCCAUCCUACCC | UUGUUGGUUGGAUGAUGGUGA | Cleavage |
| tae-miR9676-5p | TaTrx4-1B |  | 689 | 710 | UGGAUGUCAUCGUGGCCGUACA | GCUAUGGCUAUUGUGAUAUCCC | Translation |

**Table S7** | Details of subcellular localization of Trx proteins.

| **Protein accession** | **GO-id** | **GO TERM** | **Score** |
| --- | --- | --- | --- |
| TaTrx-1 | [GO:0005737](https://www.ebi.ac.uk/QuickGO/term/GO:0005737) | Cytoplasm | 0.7 |
| TaTrx-2 | [GO:0005615](https://www.ebi.ac.uk/QuickGO/term/GO:0005615) | Extracellular space | 0.91 |
| TaTrx-3 | [GO:0005737](https://www.ebi.ac.uk/QuickGO/term/GO:0005737) | Cytoplasm | 0.7 |
| TaTrx-4 | [GO:0009507](https://www.ebi.ac.uk/QuickGO/term/GO:0009507) | Chloroplast | 1 |
| TaTrx-5 | [GO:0005634](https://www.ebi.ac.uk/QuickGO/term/GO:0005634) | Nucleus | 1 |
| TaTrx-6 | [GO:0005737](https://www.ebi.ac.uk/QuickGO/term/GO:0005737) | Cytoplasm | 0.7 |
| TaTrx-7 | [GO:0005615](https://www.ebi.ac.uk/QuickGO/term/GO:0005615) | Extracellular space | 1 |
| TaTrx-8 | [GO:0005634](https://www.ebi.ac.uk/QuickGO/term/GO:0005634) | Nucleus | 1 |
| TaTrx-9 | [GO:0005737](https://www.ebi.ac.uk/QuickGO/term/GO:0005737) | Cytoplasm | 0.7 |
| TaTrx-10 | [GO:0005737](https://www.ebi.ac.uk/QuickGO/term/GO:0005737) | Cytoplasm | 0.7 |
| TaTrx-11 | [GO:0005634](https://www.ebi.ac.uk/QuickGO/term/GO:0005634) | Nucleus | 1 |
| TaTrx-12 | [GO:0005739](https://www.ebi.ac.uk/QuickGO/term/GO:0005739) | Mitochondrion | 0.7 |
| TaTrx-13 | [GO:0005634](https://www.ebi.ac.uk/QuickGO/term/GO:0005634) | Nucleus | 1 |
| TaTrx-14 | [GO:0005634](https://www.ebi.ac.uk/QuickGO/term/GO:0005634) | Nucleus | 1 |
| TaTrx-15 | [GO:0009707](https://www.ebi.ac.uk/QuickGO/term/GO:0009707) | Chloroplast outer membrane | 0.74 |

**Table S8** | Details of structural properties of TaTrx proteins.

| **S No.** | **Protein** | **Sequence identity %** | **Q mean** | **Template** | **Template description** | **Oligo state** | **Ramachandran favoured %** |
| --- | --- | --- | --- | --- | --- | --- | --- |
| 1 | TaTrx1-1A | 64.62 | 0.11 | [3d22.1.A](https://swissmodel.expasy.org/templates/3d22.1) | Thioredoxin H-type | Monomer | 99.21 |
| 2 | TaTrx2-1A | 95.41 | -0.06 | [2iwt.1.A](https://swissmodel.expasy.org/templates/2iwt.1) | Thioredoxin h isoform 2 | Hetero-dimer | 96.23 |
| 3 | TaTrx3-1B | 64.62 | 0.11 | [3d22.1.A](https://swissmodel.expasy.org/templates/3d22.1) | Thioredoxin H-type | Monomer | 99.21 |
| 4 | TaTrx4-1B | 95.41 | -0.06 | [2iwt.1.A](https://swissmodel.expasy.org/templates/2iwt.1) | Thioredoxin h isoform 2 | Hetero-dimer | 96.23 |
| 5 | TaTrx5-1B | 62.96 | -0.34 | [2iwt.1.A](https://swissmodel.expasy.org/templates/2iwt.1) | Thioredoxin h isoform 2 | Hetero-dimer | 97.17 |
| 6 | TaTrx6-1D | 64.62 | 0.11 | [3d22.1.A](https://swissmodel.expasy.org/templates/3d22.1) | Thioredoxin H-type | Monomer | 99.21 |
| 7 | TaTrx7-1D | 95.45 | 0.64 | [2vlt.1.A](https://swissmodel.expasy.org/templates/2vlt.1) | Thioredoxin h isoform 2 | Monomer | 97.22 |
| 8 | TaTrx8-1D | 63.89 | -0.17 | [2iwt.1.A](https://swissmodel.expasy.org/templates/2iwt.1) | Thioredoxin h isoform 2 | Hetero-dimer | 97.17 |
| 9 | TaTrx9-2A | 97.44 | 0.1 | [2vm1.3.A](https://swissmodel.expasy.org/templates/2vm1.3) | Thioredoxin h isoform 1 | Monomer | 99.09 |
| 10 | TaTrx10-2D | 97.44 | -3.2 | [2vm1.3.A](https://swissmodel.expasy.org/templates/2vm1.3) | Thioredoxin h isoform 1 | Monomer | 87.50 |
| 11 | TaTrx11-5A | 48.21 | -0.75 | [2vm1.3.A](https://swissmodel.expasy.org/templates/2vm1.3) | Thioredoxin h isoform 1 | Monomer | 96.33 |
| 12 | TaTrx12-5B | 61.9 | -0.2 | [1fb0.1.A](https://swissmodel.expasy.org/templates/1fb0.1) | Thioredoxin m | Monomer | 99.03 |
| 13 | TaTrx13-5B | 48.65 | -0.23 | [2vm1.3.A](https://swissmodel.expasy.org/templates/2vm1.3) | Thioredoxin h isoform 1 | Monomer | 96.30 |
| 14 | TaTrx14-5D | 48.65 | -0.25 | [2vm1.3.A](https://swissmodel.expasy.org/templates/2vm1.3) | Thioredoxin h isoform 1 | Monomer | 96.30 |
| 15 | TaTrx15-3B | 70.75 | 0.56 | [1faa.1.A](https://swissmodel.expasy.org/templates/1faa.1) | Thioredoxin f | Monomer | 96.15 |

**Table S9** | Results of Ordinary One-way ANOVA and Sidak's multiple comparisons test of gene expression and enzyme/metabolite observations.

| **Sidak's multiple comparisons test** | **TaTrx1** | | **TaTrx2** | | **TaTrx3** | | | | | **TaTrx4** | | | | | **TaTrx5** | | | | | | **TaTrx6** | | | | |
| --- | --- | --- | --- | --- | --- | --- | --- | --- | --- | --- | --- | --- | --- | --- | --- | --- | --- | --- | --- | --- | --- | --- | --- | --- | --- |
|  | **Summary** | **Adjusted P Value** | **Summary** | **Adjusted P Value** | **Summary** | | **Adjusted P Value** | | | **Summary** | | **Adjusted P Value** | | | | **Summary** | | **Adjusted P Value** | | | | **Summary** | | **Adjusted P Value** | |
| CS-0 vs. TR-0 | ns | >0.9999 | ns | >0.9999 | ns | | >0.9999 | | | ns | | >0.9999 | | | | ns | | >0.9999 | | | | ns | | >0.9999 | |
| CS-0 vs. CS-24 | * | 0.0347 | **** | <0.0001 | **** | | <0.0001 | | | *** | | 0.0001 | | | | **** | | <0.0001 | | | | *** | | 0.0001 | |
| CS-0 vs. TR-24 | **** | <0.0001 | *** | 0.0008 | *** | | 0.0006 | | | ns | | 0.9995 | | | | **** | | <0.0001 | | | | ns | | 0.2029 | |
| CS-0 vs. CS-72 | **** | <0.0001 | **** | <0.0001 | **** | | <0.0001 | | | ns | | 0.2643 | | | | **** | | <0.0001 | | | | *** | | 0.0002 | |
| CS-0 vs. TR-72 | **** | <0.0001 | ** | 0.0016 | **** | | <0.0001 | | | ns | | >0.9999 | | | | **** | | <0.0001 | | | | *** | | 0.0009 | |
| CS-0 vs. CS-144 | *** | 0.0001 | *** | 0.0004 | **** | | <0.0001 | | | ns | | >0.9999 | | | | **** | | <0.0001 | | | | *** | | 0.0002 | |
| CS-0 vs. TR-144 | ** | 0.0014 | * | 0.0102 | **** | | <0.0001 | | | ns | | 0.9991 | | | | **** | | <0.0001 | | | | *** | | 0.0003 | |
| **Ordinary One-way ANOVA result** | **P value** | <0.0001 | **P value** | <0.0001 | ns | | >0.9999 | | | **P value** | | <0.0001 | | | | **P value** | | <0.0001 | | | | **P value** | | <0.0001 | |
|  | **summary** | **** | **summary** | **** | **summary** | | *** | | | **summary** | | **** | | | | **summary** | | **** | | | | **summary** | | **** | |
| **Sidak's multiple comparisons test** | **TaTrx7** | | **TaTrx8** | | **TaTrx9** | | | | | **TaTrx11** | | | | | | **TaTrx12** | | | | | | **TaTrx13** | | | |
|  | **Summary** | **Adjusted P Value** | **Summary** | **Adjusted P Value** | **Summary** | | **Adjusted P Value** | | | **Summary** | | **Adjusted P Value** | | | | **Summary** | | **Adjusted P Value** | | | | **Summary** | | **Adjusted P Value** | |
| CS-0 vs. TR-0 | ns | >0.9999 | ns | >0.9999 | ns | | >0.9999 | | | ns | | >0.9999 | | | | ns | | >0.9999 | | | | ns | | >0.9999 | |
| CS-0 vs. CS-24 | **** | <0.0001 | * | 0.0453 | **** | | <0.0001 | | | ** | | 0.0042 | | | | **** | | <0.0001 | | | | **** | | <0.0001 | |
| CS-0 vs. TR-24 | **** | <0.0001 | ns | >0.9999 | **** | | <0.0001 | | | **** | | <0.0001 | | | | **** | | <0.0001 | | | | ns | | 0.9955 | |
| CS-0 vs. CS-72 | **** | <0.0001 | ns | 0.9503 | **** | | <0.0001 | | | * | | 0.0138 | | | | **** | | <0.0001 | | | | **** | | <0.0001 | |
| CS-0 vs. TR-72 | **** | <0.0001 | ns | >0.9999 | **** | | <0.0001 | | | **** | | <0.0001 | | | | **** | | <0.0001 | | | | **** | | <0.0001 | |
| CS-0 vs. CS-144 | **** | <0.0001 | ns | >0.9999 | **** | | <0.0001 | | | ns | | >0.9999 | | | | **** | | <0.0001 | | | | **** | | <0.0001 | |
| CS-0 vs. TR-144 | **** | <0.0001 | ns | >0.9999 | **** | | <0.0001 | | | ** | | 0.0059 | | | | **** | | <0.0001 | | | | **** | | <0.0001 | |
| **Ordinary One-way ANOVA result** | **P value** | <0.0001 | **P value** | 0.0617 | **P value** | | <0.0001 | | | **P value** | | <0.0001 | | | | **P value** | | 0.0095 | | | | **P value** | | 0.0024 | |
|  | **summary** | **** | **summary** | ns | **summary** | | **** | | | **summary** | | **** | | | | **summary** | | ** | | | | **summary** | | ** | |
| **Sidak's multiple comparisons test** | **TaTrx14** | | **TaTrx15** | |  | | | | |  | | | | | |  | | | | | |  | | | |
|  | **Summary** | **Adjusted P Value** | **Summary** | **Adjusted P Value** |  |  |  |  |  |  |  |  |  |  |  |  |  |  |  |  |  |  |  |  |  |
| CS-0 vs. TR-0 | ns | >0.9999 | ns | >0.9999 |  |  |  |  |  |  |  |  |  |  |  |  |  |  |  |  |  |  |  |  |  |
| CS-0 vs. CS-24 | **** | <0.0001 | **** | <0.0001 |  |  |  |  |  |  |  |  |  |  |  |  |  |  |  |  |  |  |  |  |  |
| CS-0 vs. TR-24 | **** | <0.0001 | **** | <0.0001 |  |  |  |  |  |  |  |  |  |  |  |  |  |  |  |  |  |  |  |  |  |
| CS-0 vs. CS-72 | **** | <0.0001 | **** | <0.0001 |  |  |  |  |  |  |  |  |  |  |  |  |  |  |  |  |  |  |  |  |  |
| CS-0 vs. TR-72 | **** | <0.0001 | **** | <0.0001 |  |  |  |  |  |  |  |  |  |  |  |  |  |  |  |  |  |  |  |  |  |
| CS-0 vs. CS-144 | **** | <0.0001 | **** | <0.0001 |  |  |  |  |  |  |  |  |  |  |  |  |  |  |  |  |  |  |  |  |  |
| CS-0 vs. TR-144 | **** | <0.0001 | **** | <0.0001 |  |  |  |  |  |  |  |  |  |  |  |  |  |  |  |  |  |  |  |  |  |
| **Ordinary One-way ANOVA result** | **P value** | 0.0216 |  | 0.0005 |  |  |  |  |  |  |  |  |  |  |  |  |  |  |  |  |  |  |  |  |  |
|  | **summary** | * |  | *** |  |  |  |  |  |  |  |  |  |  |  |  |  |  |  |  |  |  |  |  |  |
| **Sidak's multiple comparisons test** | **CAT** | | **POX** | | **HP** | | | | **SOR** | | | | | **SOD** | | | | | | **APX** | | | | |  |
|  | **Summary** | **Adjusted P Value** | **Summary** | **Adjusted P Value** | **Summary** | **Adjusted P Value** | | **Summary** | | | **Adjusted P Value** | | **Summary** | | | | **Adjusted P Value** | | **Summary** | | | | **Adjusted P Value** | |  |
| CS-0 vs. TR-0 | ns | >0.9999 | ns | <0.0001 | ns | 0.6179 | | ns | | | 0.9454 | | ns | | | | 0.9466 | | ns | | | | 0.9794 | |  |
| CS-0 vs. CS-72 | ** | 0.0065 | **** | 0.9308 | **** | <0.0001 | | **** | | | <0.0001 | | ** | | | | 0.0013 | | **** | | | | <0.0001 | |  |
| CS-0 vs. TR-72 | ** | 0.0014 | **** | 0.0016 | ns | 0.5933 | | **** | | | <0.0001 | | **** | | | | <0.0001 | | **** | | | | <0.0001 | |  |
| **Ordinary One-way ANOVA result** | **P value** | 0.0007 | **P value** | <0.0001 | **P value** | <0.0001 | | **P value** | | | <0.0001 | | **P value** | | | | <0.0001 | | **P value** | | | | <0.0001 | |  |
|  | **summary** | *** | **summary** | **** | **summary** | **** | | **summary** | | | **** | | **summary** | | | | **** | | **summary** | | | | **** | |  |
